# Effects of Three Motivationally Targeted Mobile Device Applications on Initial Physical Activity and Sedentary Behavior Change in Midlife and Older Adults: A Randomized Trial

**DOI:** 10.1371/journal.pone.0156370

**Published:** 2016-06-28

**Authors:** Abby C. King, Eric B. Hekler, Lauren A. Grieco, Sandra J. Winter, Jylana L. Sheats, Matthew P. Buman, Banny Banerjee, Thomas N. Robinson, Jesse Cirimele

**Affiliations:** 1 Stanford Prevention Research Center, Department of Medicine, and Epidemiology Division, Department of Health Research & Policy, Stanford University School of Medicine, Stanford, California, United States of America; 2 Stanford Prevention Research Center, Department of Medicine, Stanford University School of Medicine, Stanford, California, United States of America; 3 School of Earth, Energy and Environmental Sciences, Stanford University, Stanford, California, United States of America; 4 Department of Pediatrics, Stanford University School of Medicine, Stanford, California, United States of America; 5 Department of Computer Science, School of Engineering, Stanford University, Stanford, California, United States of America; University of Tennessee Health Science Center, UNITED STATES

## Abstract

**Background:**

While there has been an explosion of mobile device applications (apps) promoting healthful behaviors, including physical activity and sedentary patterns, surprisingly few have been based explicitly on strategies drawn from behavioral theory and evidence.

**Objective:**

This study provided an initial 8-week evaluation of three different customized physical activity-sedentary behavior apps drawn from conceptually distinct motivational frames in comparison with a commercially available control app.

**Study Design and Methods:**

Ninety-five underactive adults ages 45 years and older with no prior smartphone experience were randomized to use an analytically framed app, a socially framed app, an affectively framed app, or a diet-tracker control app. Daily physical activity and sedentary behavior were measured using the smartphone’s built-in accelerometer and daily self-report measures.

**Results:**

Mixed-effects models indicated that, over the 8-week period, the social app users showed significantly greater overall increases in weekly accelerometry-derived moderate to vigorous physical activity relative to the other three arms (*P* values for between-arm differences = .04-.005; Social vs. Control app: d = 1.05, CI = 0.44,1.67; Social vs. Affect app: d = 0.89, CI = 0.27,1.51; Social vs. Analytic app: d = 0.89, CI = 0.27,1.51), while more variable responses were observed among users of the other two motivationally framed apps. Social app users also had significantly lower overall amounts of accelerometry-derived sedentary behavior relative to the other three arms (*P* values for between-arm differences = .02-.001; Social vs. Control app: d = 1.10,CI = 0.48,1.72; Social vs. Affect app: d = 0.94, CI = 0.32,1.56; Social vs. Analytic app: d = 1.24, CI = 0.59,1.89). Additionally, Social and Affect app users reported lower overall sitting time compared to the other two arms (*P* values for between-arm differences < .001; Social vs. Control app: d = 1.59,CI = 0.92, 2.25; Social vs. Analytic app: d = 1.89,CI = 1.17, 2.61; Affect vs. Control app: d = 1.19,CI = 0.56, 1.81; Affect vs. Analytic app: d = 1.41,CI = 0.74, 2.07).

**Conclusion:**

The results provide initial support for the use of a smartphone-delivered social frame in the early induction of both physical activity and sedentary behavior changes. The information obtained also sets the stage for further investigation of subgroups that might particularly benefit from different motivationally framed apps in these two key health promotion areas.

**Trial Registration:**

ClinicalTrials.gov NCT01516411

## Introduction

Current advances in mobile communication technology have paved the way for an explosion of digital health promotion programs and mobile applications (apps), many aimed at promoting physical activity [1–3]. Mobile apps targeting health behavior change hold great promise with respect to offering customized information, continuity of information and support over time, and a potentially wide reach across virtually the entire population. This includes the increasingly prevalent midlife and older segments of the population (ages 45 years and above) that are at increasing risk for the range of chronic diseases that are positively impacted by regular physical activity, but represent the most inactive age group in the U.S. and elsewhere [4]. This age group stands to benefit greatly from the rapid growth of smartphone usage and accompanying health apps. In 2015, about half of adults ages 45–64 years owned a smartphone, along with a 42% increase in smartphone ownership between 2014 and 2015 in adults ages 65 and over (to 27% in 2015) [5]. In addition, 52% of U.S. adults with a high school education or less and 50% of adults in the lowest third of household income now own smartphones.

While thousands of health promoting smartphone apps are currently available, relatively few have applied behavioral theory in a systematic or explicit way [6, 7], and most have not been evaluated for even short-term efficacy or scientific accuracy [8, 9]. Similarly, few apps and other eHealth or mHealth programs have been aimed at bridging the “digital divide” through incorporating elements that would enable use by typically underserved populations with generally lower levels of technology literacy as well as physical activity, e.g., aging adults [10]. Direct, head-to-head comparisons of mobile apps for health behavior change have been rare, and relatively few studies in this field have evaluated more than one health behavior target at a time [11], or included less studied behaviors, such as sedentary time (i.e., seated/reclining with low energy expenditure) [12]. Evaluation of mobile apps that provide integrated adaptive information throughout the day on two complementary health behaviors (i.e., physical activity and sedentary behavior) has rarely occurred.

The purpose of this investigation was to evaluate systematically, using a controlled experimental design, the initial efficacy of three customized smartphone applications, relative to a commercially available dietary control app, on initial adoption of a more physically active and less sedentary pattern of behavior throughout the day in a sample of insufficiently active midlife and older adults who were not regular smartphone users. These “move more, sit less” apps each focused on a distinct behaviorally based motivational “frame” to better understand their relative efficacy in a sample of midlife and older adults with little mobile technology experience. This older inactive population, while carrying greater chronic disease burden than their younger counterparts, has rarely been targeted specifically in the mobile app health promotion arena [6].

The primary questions of interest were:

- Would each of the three custom apps produce significant improvements in moderate-to-vigorous physical activity (MVPA) and/or sedentary behavior relative to the control app during the eight-week study period? And
- Would any of the three custom apps produce significant eight-week improvements in MVPA and/or sedentary behavior relative to either of the other custom apps?

## Methods

### Ethics Statement

The Stanford University School of Medicine Human Subjects Institutional Review Board approved this study in September 2009. All participants provided written informed consent.

### Trial Design and Participants

The study was a parallel, randomized trial with 1:1 allocation of participants to use one of three custom physical activity apps or a diet-tracking control app for an 8-week period (described below). Random assignment occurred through use of a computerized version of the Efron procedure [13] by the study statistical analyst, who was blinded to participant allocation assignment. The Efron procedure, while not guaranteeing identical final participant allocation numbers across study arms, allows for reasonably balanced subject allocation throughout the entire recruitment and randomization period, which is particularly advantageous for studies with smaller sample sizes and multiple study arms [13]. Enrollment and intervention study assignment procedures were carried out by trained, PhD-level study staff.

The target population consisted of community-dwelling adults who met the following eligibility criteria: ages 45 years and older; insufficiently physically active (i.e., engaged in less than 60 minutes of moderate or more vigorous physical activity per week that increased heart rate, breathing, or perspiration); reported typically sitting for 10 or more hours per day; were able to participate safely in a physical activity program based on responses to the physical activity readiness questionnaire [14]; and were currently using a mobile phone though not a smartphone. The rationale for recruiting individuals who were not current smartphone users was twofold: 1) it facilitated the development of apps that were sufficiently simple and straightforward that they could be used regardless of smartphone proficiency; and 2) it simplified uniform testing of the Android apps in this first-generation study through using a standard smartphone model and platform, which also eliminated the need for participants to carry more than one phone with them or temporarily use a smartphone different from the one that they already had. The primary recruitment methods used were email invitations sent to local electronic mailing lists; in-person recruitment activities occurring at local community and senior centers as well as congregate housing settings; flyers and posters on community notice-boards; and ads on Craigslist.com. After suitable iterative design development and formative testing of the apps, Wave 1 of the study (n = 36) was conducted between April and December of 2011 to confirm the acceptability and feasibility of the study procedures and protocols for the smartphone-naïve, older population being targeted. With the acceptability of the study procedures thus confirmed, the study was registered at ClinicalTrials.gov (Identifier NCT01516411) on 19 January, 2012 and the second wave of participants (n = 59) was enrolled between February and November, 2012. The use of the two waves also helped to ensure adequate subject enrollment across all four seasons, given the relationship between seasonality and physical activity participation [15]. Study follow-up ended as planned June 2013. A copy of the study trial protocol (S1 Protocol) is included as a supporting information file, along with the CONSORT Checklist (S1 CONSORT Checklist). Data may be requested from the corresponding author. The authors confirm that all ongoing and related trials for this intervention are registered.

### Interventions: App Development and Descriptions

A detailed description of both app development and descriptions have been published elsewhere [16]. Briefly, a behavioral science-informed user experience iterative design process (BSUED) was applied in the initial development and formative testing of three different smartphone apps [17], each of which emphasized a distinct type of motivational frame [16]. The Android smartphone platform was used given its ability to provide “live wallpaper”-based glance-able displays, ease of programming, and its ability to run the built-in accelerometer in the background while other smartphone functions were operating [16]. The objective of each app was to shape the user towards a daily goal of approximately 30 minutes of moderate-to-vigorous physical activity and eight or fewer hours of daily sedentary time [18, 19], measured using the smartphone’s built-in accelerometer. To standardize the smartphones being used in this first-generation study and to mitigate potential problems that can occur when current smartphone users are asked to use an additional phone, individuals were recruited who were mobile phone users but not regular smartphone users. All participants were provided with the same model of Android smartphone.

Among the aims of the iterative design and formative user testing process were to develop custom apps that would be generally appealing and engaging for the aging population being targeted. All three apps shared basic behavioral and design elements that have been found to elicit interest and initial participation, including both “push” (notifications) and “pull” (information available via selecting an icon) components; a glance-able display; real-time customized feedback that was driven by the personal data being captured via the built-in accelerometer; behavioral self-monitoring; and a help tab [16, 17]. The above components provided each participant with his/her own customized “just-in-time” feedback and information based on the data being automatically collected by each participant’s smartphone throughout the day.

The specific format and content of each app are described previously [16]. Briefly, the “analytic” app, based on Social Cognitive theory and self-regulatory principles of behavior change [20, 21], emphasized personalized and quantitative goal-setting, behavioral feedback, informational tips promoting behavior change, and problem-solving strategies aimed at behavior change barriers. Two colorful meters, viewable on the phone’s glance-able display throughout the day, showed progress towards goals for moderate-to-vigorous physical activity (MVPA) and sedentary behavior.

The “social” app, based largely on social influence perspectives [22, 23], emphasized social support for behavior change, “just-in-time” social normative feedback [24], modeling of behaviors by similar others using avatars on the display, and group-based collaboration and competition. Small avatars reflecting the current physical activity/sedentary behavior levels of the participant and other members of the “virtual team” to which he/she had been automatically assigned, as well as another “virtual team”, were viewable on the phone’s glance-able display throughout the day. Team members could also interact with one another through the app’s online message board.

The “affect” app, which applied principles of reinforcement scheduling, attachment and nurturance motives, as well as gamification/play [16], utilized an avatar in the form of a bird to mirror how active or sedentary the user was throughout the day. The bird avatar, which was viewable on the phone’s glance-able display throughout the day, changed position, posture, and movement depending on how active or inactive the user was up to that time point. Users received “rewards” (e.g., the bird avatar would unexpectedly appear in far-away cities) as a function of increased physical activity levels.

Following the iterative design development and formative testing process (of both the user experience and app functionality), we conducted the first-generation randomized controlled experiment of the three apps evaluating their capabilities for impacting initial (2-month) physical activity and sedentary activity levels, measured via smartphone-based accelerometry, which was validated against Actigraph accelerometry [25], relative to a commercially available, non-physical activity control app (Calorific), which tracks dietary behaviors throughout the day. As noted earlier, individuals meeting the eligibility criteria were randomly assigned to use one of the three custom apps or the diet-tracking control app for an 8-week period. The first week of the 8-week period was used as a baseline period during which time only the activity-monitoring app (without a behavior change app) was installed on the study smartphones provided to participants. Participants were requested to continue their normal physical activity and sedentary behaviors during the baseline week. Because the participants in this study had not previously used a smartphone, an initial one-on-one 1-hour training session was used to provide participants with instruction on the general use of the smartphone, including wearing it attached to their waist to optimize accurate data capture via the phone’s built-in accelerometer. At the end of this initial week, participants returned to the research facility to receive their randomly assigned behavior change app and basic instruction on its use. In addition to having access to the ‘‘help” tab as part of each app, participants could call project staff with any technical problems or difficulties with the apps during the 8-week project period. In addition, written instructions for the smartphone were provided in the form of the manufacturer’s user manual along with simplified user instructions designed by the research team to highlight key features of both the smartphone and the apps.

### Measures

#### Accelerometry-derived daily activity and sedentary time

The primary outcome measure for this study was estimated minutes in moderate-to-vigorous intensity physical activity (MVPA) based on smartphone-derived accelerometry. Secondary outcome measures were estimated minutes of sedentary time based on smartphone-derived accelerometry and self-reported ecological momentary assessment of daily brisk walking and sitting time collected via the smartphone. We utilized the cut-points generated from our prior research to determine MVPA and sedentary time (see below). These data were collected via the smartphones themselves and sent wirelessly to a secured Stanford University server for storage and analysis. The built-in smartphone accelerometer was used because of this older, technology-naïve population’s general reluctance, revealed during the piloting phase of the study, to wear or carry more than one electronic device.

Smartphone accelerometry data collection and cleaning procedures were consistent with large-scale cross-sectional accelerometer studies [26, 27], such that: (a) valid hours of data consisted of no more than 60 consecutive ‘zero’ values (interpreted as non-wear time); and (b) a valid day was defined as at least 10 valid hours/day. Because data were collected continuously and trajectory-based analyses were conducted, no minimum valid days/week criterion was necessary. As noted above, minutes of moderate-vigorous (>301 counts/min) and sedentary (≤56 counts/min) activities using the phone’s built-in accelerometer were calculated based on our prior validation work using this type of smartphone accelerometer [25].

#### Ecological Momentary Assessment (EMA) of target health behaviors

In addition to collecting movement data throughout the day via the smartphone’s built-in accelerometer, ecological momentary assessment-based (EMA) reporting of brisk walking and sitting time was also collected on a daily basis via the smartphone throughout the 8-week study period. EMA, a method whereby individuals are queried on behaviors, affect, and similar events close in time to the experience of such events, can facilitate more sensitive and accurate recording and/or prediction of such events or behaviors [28, 29]. Daily reporting occurred each evening, at a time most convenient to each participant. In the event that a participant failed to respond at the set time, automated reminder notifications were sent to the smartphone. The brisk walking item consisted of the following question: “How many total minutes of brisk walking have you engaged in today? Choose one response”. Categories of total minutes were as follows: 1–9, 10–14, 15–19, 20–24, 25–29, 30–34, 35–39, 40–49, 50–59, 60–74, 75–89, 90–119, and more than 120 minutes. The sitting time item consisted of the following question: “How many total hours have you been sitting today?” Participants chose a number between 0 and 24 hours.

### Statistical Analyses

Sample size calculations for this first-generation smartphone intervention study were based on a prior eight-week physical activity mobile device intervention study with similarly aged insufficiently active adults [30]. In that study, between-arm differences were achieved with approximately 18 participants per arm completing the study. The sample size was increased by approximately 22 percent per arm in the current trial to allow for multiple testing, in conjunction with adding objective measurement of the two major outcome variables (physical activity and sedentary behavior) and use of mixed models analysis to enhance statistical power.

Mixed models analyses were utilized because of the intensive repeated measures design [31]. The advantages of this technique are that it is capable of handling nested observations (e.g., multiple observations for each individual), unbalanced (i.e., unequal) numbers of observations, and missing values [32]. Physical activity (i.e., accelerometer-derived moderate-to-vigorous physical activity, EMA-derived brisk walking) and sedentary behavior (i.e., accelerometer-derived sedentary time, EMA-derived sitting time) outcomes were entered as dependent variables in separate models. Wave of study recruitment and linear, quadratic, and cubic time parameterizations were adjusted for in all models. Main effects for time, and time x study arm interactions were tested. Full-information maximum likelihood estimation was used as part of SAS Enterprise Guide v5.1 software to accommodate missing data in the models. To provide more easily interpretable data, we centered our time variable to the last baseline date (i.e., intervention day 1 = 1), thus allowing easier interpretation of the results. Because of a non-normal distribution of MVPA estimates, MVPA values were log-transformed. Scheffé adjustment for multiple comparisons—customarily used with unequal cell sizes—was applied in interpreting differences between pairs of study arms. The significance level for all statistical analyses was set at *P* < .05, two-tailed.

## Results

### Participant Sample

**Fig 1:**
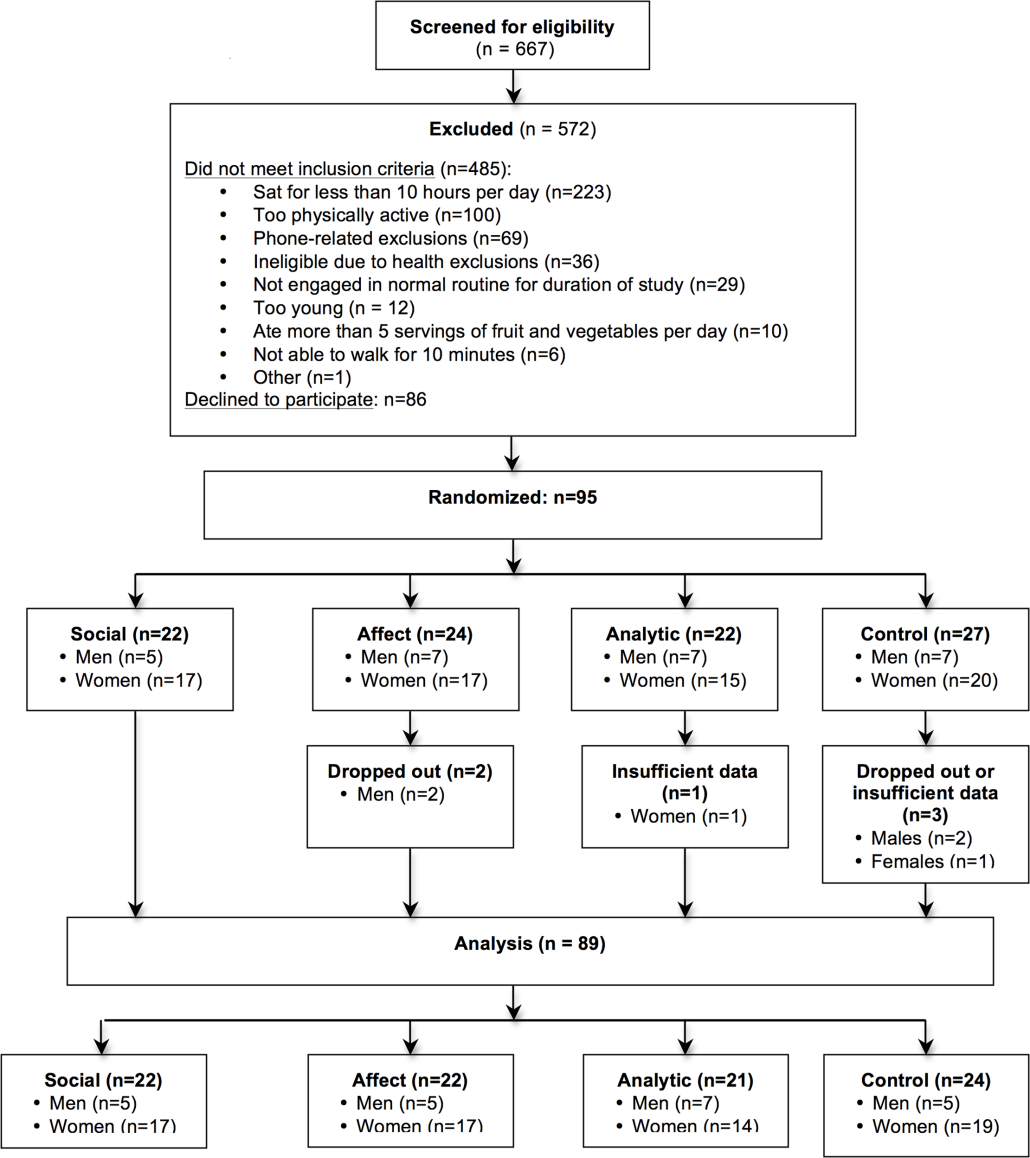
Study Enrollment and Retention Flowchart.

Study enrollment, flow, and number of participants per arm that were included in data analyses are summarized in **Fig 1**. In brief, approximately one fourth of the sample was randomized to each smartphone app (Social n = 22; Affect n = 24; Analytic n = 22, Control = 27). A slightly higher number of participants were randomized to the Control arm, given the potential for somewhat greater dropout occurring with the diet-tracker app used in that arm. The overall study retention rate equaled 94%, with no significant differences between study arms (retention rates for Social = 100% [22/22]; Affect = 92% [22/24]; Analytic = 95% [21/22]; Control = 89% [24/27]). **Table 1** reports sample descriptive statistics. No significant between-group baseline differences were found for the demographic variables, physical activity, or sedentary behavior variables measured via accelerometry or ecological momentary assessment (*P* values ≥ .15). All analyses were conducted using original group assignment. No adverse events or harms were found across the study period.

**Table 1:** Descriptive Variables, by Arm.

	**All**		**Affect**		**Analytic**		**Social**		**Control**	
Variable (categorical)	**N**	**%**	**N**	**%**	**N**	**%**	**N**	**%**	**N**	**%**
Gender										
Female	67	75.3	17	77.3	14	66.7	17	77.3	19	79.2
Male	22	24.7	5	22.7	7	33.3	5	22.7	5	20.8
Race										
White	75	84.3	18	81.8	19	90.5	18	81.8	20	83.3
Asian	10	11.2	1	4.6	1	4.7	4	18.2	4	16.72
Other	2	2.2	1	4.6	1	4.7	0	0.0	0	0.0
No response	2	2.2	2	9.0	0	0.0	0	0.0	0	0.0
Marital status										
Married	40	44.9	6	27.3	9	42.8	10	45.4	15	62.5
Separated/divorced	16	18.0	5	22.7	5	23.8	3	13.6	3	12.5
Widowed	8	9.0	1	4.5	1	4.8	1	4.6	5	20.8
Single	21	23.6	8	36.4	5	23.8	7	31.8	1	4.2
Other	4	4.5	2	9.1	1	4.8	1	4.6	0	0.0
Education										
High school	2	2.3	1	4.6	0	0.0	0	0.0	1	4.2
Some college	19	21.3	2	9.1	7	33.3	6	27.3	4	16.7
Bachelor degree	34	38.2	12	54.5	3	14.3	11	50.0	8	33.3
Master degree	23	25.8	4	18.2	7	33.3	4	18.2	8	33.3
Doctoral degree	11	12.4	3	13.6	4	19.1	1	4.5	3	12.5
Household income										
<20,000	7	7.9	1	4.6	2	9.5	3	13.6	1	4.2
20,000–39,000	8	9.0	3	13.6	1	4.8	2	9.1	2	8.3
40,000–59,000	9	10.1	4	18.2	2	9.5	3	13.6	0	0.0
60,000–79,000	16	18.0	4	18.2	4	19.0	3	13.6	5	20.8
80,000–99,000	10	11.2	1	4.6	3	14.3	2	9.1	4	16.7
>100,000	34	38.2	7	31.8	9	42.9	8	36.4	10	41.7
No response	5	5.6	2	9.0	0	0.0	1	4.6	2	8.3
	**All**		**Affect**		**Analytic**		**Social**		**Control**	
Variable (continuous)	Mean	SD	Mean	SD	Mean	SD	Mean	SD	Mean	SD
Age (yr.)	60.0	9.3	59.5	10.0	59.5	9.5	57.9	7.7	62.8	9.8
Body Mass Index (kg/m^2^)	28.8	6.0	28.9	7.9	29.7	4.7	30.2	6.2	26.7	4.7
Number in household	2.3	1.3	1.9	0.9	2.2	1.3	2.6	1.3	2.5	1.6
Baseline accelerometry sedentary activity (min/day)	459.5	86.9	452.8	115.3	467.9	93.5	440.4	53.3	474.0	74.2
Baseline accelerometry mod. /vigorous activity (min/day)	21.5	17.9	26.8	25.6	18.2	15.5	22.7	13.0	18.5	14.4
Baseline EMA derived brisk walking (min/day)	19.1	18.4	19.6	20.1	21.3	16.4	16.1	21.4	19.4	15.5
Baseline EMA derived sitting time (hr)day)	8.5	2.4	8.3	1.7	8.8	1.8	8.5	3.1	8.4	3.0

^a^EMA = Ecological Momentary Assessment

### Changes in Accelerometer-derived Moderate-to-Vigorous Physical Activity (MVPA)

**Fig 2:**
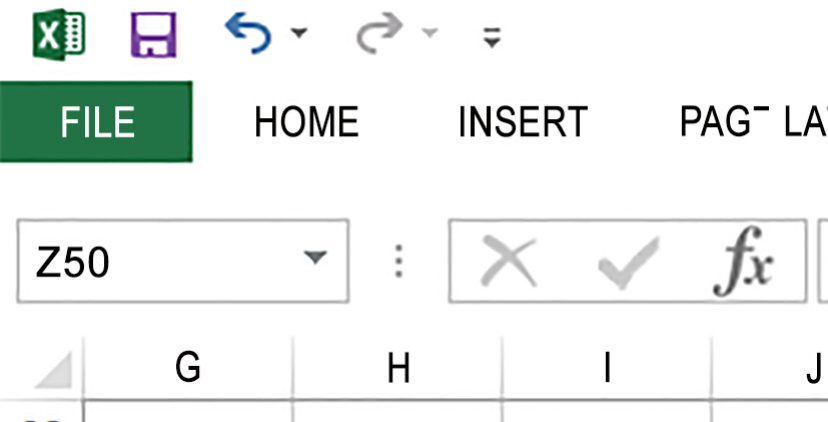
Changes in Accelerometer-Derived MVPA by Study Arm.

**Fig 2** graphically displays overall changes in accelerometer-derived MVPA by study arm. Overall, there were significant quadratic (F[1,2647] = 18.63, *P* < .001) and cubic (F[1,2647] = 8.00, *P* = .005) effects for time. The primary questions of interest pertained to whether any of the customized apps would promote significantly higher 8-week MVPA levels relative to the Control app, and whether the effects of the customized apps in promoting 8-week MVPA would differ significantly from one another. Significant main effect differences over time were observed by study arm such that the Social app had significantly higher levels of MVPA relative to the Control app (mean difference [SE] = 3.17 [0.02]; t = 3.57, *P* = .005; d = 1.05, CI = 0.44,1.67), the Affect app (mean difference [SE] = 1.38 [.02];t = 2.94, *P* = .03; d = 0.89, CI = 0.27,1.51), and the Analytic (mean difference [SE] = 1.27 [.02]; t = 2.84,*P* = .04; d = 0.89, CI = 0.27,1.51) following Scheffé adjustment for multiple comparisons. No other significant between-arm differences were found and no time x arm differences were observed. Based upon a significant random effect for time (*P* < .001), there was substantial amount of inter-individual variability in changes in MVPA across all of the apps.

### Changes in Accelerometer-Derived Sedentary Behavior

**Fig 3:**
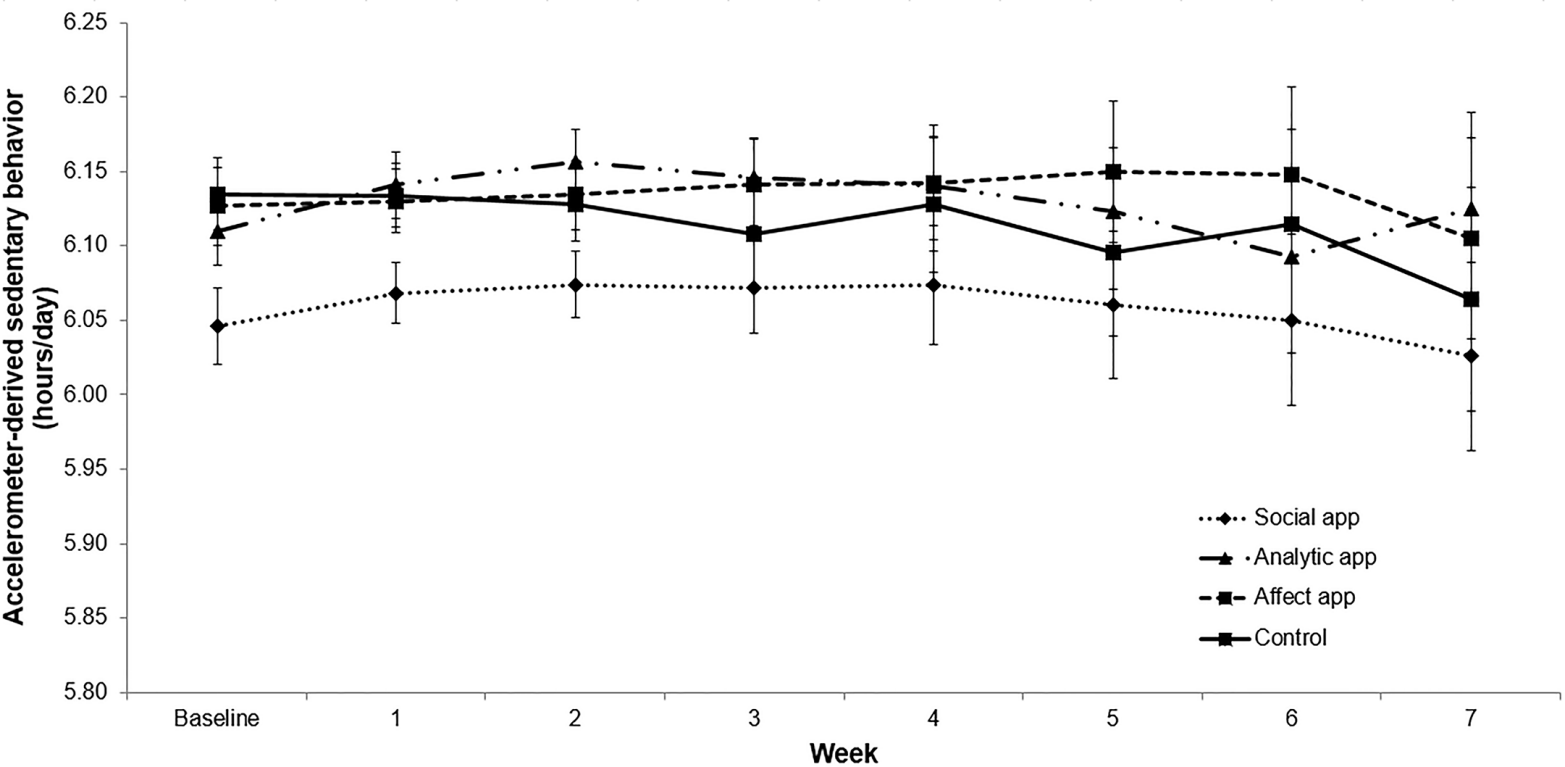
Changes in Accelerometer-derived Sedentary Behavior by Study Arm.

**Fig 3** graphically displays overall changes in accelerometer-derived sedentary behavior by study arm. Overall, there were non-significant linear, quadratic, and cubic effects for time (*P* values = .10-.24). However, significant main effect differences over time were observed by study arm, similar to those observed for MVPA. The Social app had significantly lower levels of sedentary time relative to the Control app (mean difference [SE] = -.05 [.01]; t = -3.72, *P* < .001; d = 1.10,CI = 0.48,1.72), the Affect app (mean difference [SE] = -.09 [.01];t = -3.11, *P* = .02; d = 0.94, CI = 0.32,1.56), and the Analytic (mean difference [SE] = -.09 [.01]; t = -4.07,*P* < .001; d = 1.24, CI = 0.59,1.89) following Scheffé adjustment for multiple comparisons. No other significant between-arm differences were found and no time x arm differences were observed. Based upon a significant random effect for time (*P* < .001), there was substantial amount of inter-individual variability in changes in sedentary time across all of the apps.

### Changes in Ecological Momentary Assessment (EMA) Outcomes

**Fig 4:**
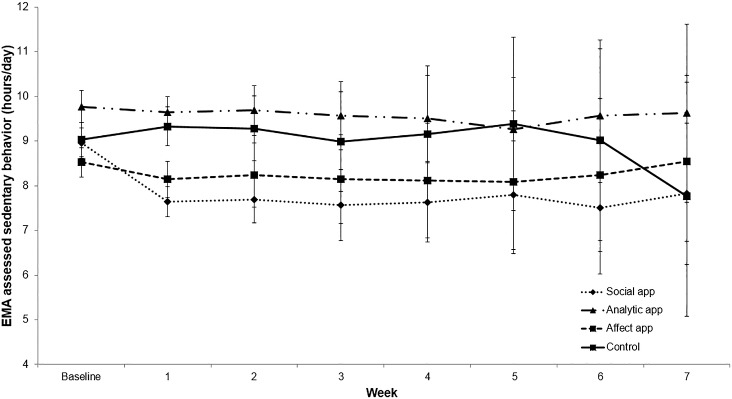
Changes in EMA-Assessed Sitting Time by Study Arm.

For the smartphone EMA-reported brisk walking variable, there were no significant overall effects for time or statistically significant differences between study arms. For reported sitting time (see **Fig 4**), there was an overall significant cubic effect for time (F[1,1264] = 6.82, *P* < .001). Significant main effect differences over time were also observed by study arm. The Social and Affect apps both reported significantly less sitting time than either the Analytic app (Social app: mean difference [SE] = 1.00 [.03];t = 6.19, *P* < .001, d = 1.89,CI = 1.17, 2.61; Affect app: mean difference [SE] = .90 [.21];t = 4.61, *P* = < .001, d = 1.41,CI = 0.74, 2.07) or the Control app (Social app: mean difference [SE] = .46 [.39];t = 5.37, *P* = < .001, d = 1.59,CI = 0.92, 2.25; Affect app: mean difference [SE] = .36 [.64];t = 4.02, *P* < .001, d = 1.19,CI = 0.56, 1.81) following Scheffé adjustment for multiple comparisons. No other significant between-arm differences were found and no time x arm differences were observed. Based upon a significant random effect for time (*P* < .001), there was substantial amount of inter-individual variability in changes in EMA-based sitting time across all of the apps.

### For the Social App: Exploration of Between-Participant Social Interactions

Given the significant effects of the Social app on both accelerometry-derived target variables and the EMA-based sitting time variable, preliminary qualitative assessment of the Social app’s message board content was conducted to gain an understanding of the types of messages that were being posted by participants [33]. During the study period, 91.3% of social app participants used the message board, with a total of 775 messages posted. Based on published behavioral intervention taxonomies [34, 35], the top five most frequently used thematic categories were: discussion of barriers to improve physical activity (79% of posted messages contained these themes); expressing gratitude, support, commitment, or affirmations to the group (64% of messages); comments on the usability of the app (64% of messages); stating achievements towards a goal (55% of messages); and providing validation and support for others’ accomplishments (48% of messages). These exploratory findings suggest that the message board was being used by the majority of Social app participants in ways that were consistent with regulatory skill-building [33].

### Post-Intervention User Satisfaction with the Three Apps

User satisfaction related to the three customized apps has been reported previously [16]. Briefly, most participants reported that the apps helped remind them (71%) and motivate them (69%) to increase their physical activity levels as well as sit less throughout the day (87% and 74%, respectively) [16]. There were no significant differences found for such satisfaction ratings between apps.

## Discussion

The results of the current study indicate that, among a sample of midlife and older adults who were insufficiently active and reported sitting for prolonged periods throughout the day, a customized smartphone app that specifically targeted social elements and contexts showed significantly greater short-term improvements in both accelerometer-based MVPA and sedentary behavior relative to a non-physical activity control app as well as the other two intervention apps that specifically targeted different motivational frames (affect/nurturance framing and analytic framing). This was the case even though all three apps were developed using extensive formative evaluation of target group interests and preferences, along with user-experience testing and iteration. The potency of the types of social influences incorporated into this app (i.e., social norm comparisons, implicit cues for within-team collaboration and between-team competition, social interaction opportunities between team members) have been noted in other evaluations of mHealth interventions for physical activity as well as other types of health behaviors [36–38]. Future studies would do well to explore the isolated effects of these different aspects of social influence and support, along with aspects of app functionality and usability, on physical activity and sedentary behavior outcomes. Of note, the overall effect size estimates for the differences found were reasonably large, suggesting potentially meaningful differences that merit further study.

While the Social app showed the largest average changes in physical activity and sedentary behavior across the 8-week study period relative to the other apps, the mean overall changes across this time period were reasonably modest and variable from week to week (as reflected in Figs 2–4). These results and those from other behavioral health interventions suggest that there are likely participant subgroups for each intervention app comprising the overall group means that deserve further systematic evaluation [39]. Unfortunately, the reasonably small sample size in the current investigation prevented the types of subgroup analyses that could have shed further light on what has been called the “whiches conundrum” (i.e., which interventions for which individuals under which circumstances) [40, 41]. Given the diversity of human preferences and experiences, such subgroup analyses are an important means of better customizing behavioral mHealth programs to optimize success (i.e., via a “behavioral” precision health paradigm).

Self-reported sitting time has been found to be associated with all-cause mortality in a growing number of (though not all [42]) observational studies as well as in a range of countries [43]. Daily self-reports of health behaviors collected via the smartphone supported the decreases in sitting represented via the accelerometry-derived information in the Social app arm, and also indicated decreases in reported sitting time among users of the Affect app. As shown in Figs 3 and 4, the overall shapes of the curves for these two different forms of sedentary behavior measurement were reasonably similar for the Affect app. It is possible that the increased variability derived via the accelerometry-based measurement reduced power to detect significant differences in that arm, given that the accelerometry-based measurement captures overall sedentary behavior, while the behavioral goal reflected in the apps was sitting time. It would be worthwhile exploring these issues further with larger sample sizes and across longer time periods.

Among the strengths of the current investigation were its focus on smartphone applications—an increasingly accessible and scalable delivery system for behavioral health interventions, yet one in which many programs have received surprisingly little systematic evaluation to determine initial efficacy; use of a formal iterative design process to optimize the applicability of the three customized apps for the target population; targeting of two health behaviors that, while complementary, have been found to have independent effects on important health outcomes [44]; measurement of both health behaviors through objective accelerometry, which also allowed the apps to provide accurate “just-in-time” feedback to users [24]; and an experimental design in which three different motivationally framed apps were compared “head-to-head” to move the field forward in an arguably more efficient way. The targeting of sedentary behavior, in particular, through this mobile communication mode has received relatively little attention to date. Additionally, while population aging and the increasing physical inactivity accompanying it are occurring globally [45], few studies in this area have specifically targeted aging adults in mobile application development and testing [46].

In addition to the above strengths, this “first-generation” intervention study contained a number of limitations that require attention in future work. These include the small sample size and the short intervention time frame which, while useful in evaluating initial app use and induction of changes in physical activity and sedentary behavior, do not systematically address the longer-term use and sustainability issues that remain a major challenge in the eHealth and mHealth fields [9, 36, 47]. Whether the currently tested apps would have been able to maintain user interest and improve the target health behaviors across longer time periods deserves further systematic investigation. To begin to gather information about the potential possibilities for sustained use of each of the apps, we conducted formative work in which 12 participants enrolled in the latter stage of the study (four from each custom app arm who were approached in consecutive order just prior to their study end date) were allowed to continue using their assigned app, if they chose, until all smartphones were collected on day 233 post-study [16]. These participants continued to use their apps for a mean of 191 [SD = 33] days post-study (range = 120–233, with sample sizes too small to explore differences between apps) [16]. These data do provide an initial positive “signal” concerning longer-term use that would be worth investigating in a systematic way.

An additional limitation concerns the use of the smartphones’ built-in accelerometer that, while shown in prior research to be a valid and reliable means of capturing physical activity and sedentary behavior, required participants to wear the phone daily. Expanding the capabilities of such apps to reliably interface with the growing number of wearable accelerometers in the marketplace are increasingly important. This point notwithstanding, it should be noted that the smartphones’ built-in accelerometer was successfully validated against what is currently considered to be the “gold standard” in research-grade ambulatory sensing devices, i.e., the Actigraph [25]. In addition, a recent investigation involving a systematic head-to-head comparison of nine wearable devices currently on the market showed that none of them exceled in accurately capturing both daily physical activity and sedentary behavior [48]. A major challenge facing the wearable device industry is to develop suitable monitors that can accurately capture all three major behaviors—activity, sedentary behavior, and sleep—occurring across the 24-hour day [48]. Similarly, the built-in accelerometer, similar to Actigraph, Fitbit, and similar devices, is not as sensitive to measuring bouts of sedentary behavior as other devices (e.g., ActivPAL, LUMOback) [48]. This circumstance limited our ability to accurately ascertain differences in numbers of breaks in daily sedentary behavior. The relatively few studies that have systematically measured the effects of breaks in daily sitting time on relevant proximal health outcomes have produced mixed results [49], and this area deserves further systematic attention.

In summary, the results from this first-generation experiment support the overall effectiveness of a socially framed app, customized to the preferences and needs of midlife and older adults with minimal smartphone experience, in the early improvement of physical activity and sedentary behavior patterns. In addition, some partial support was obtained for the further exploration of an affectively framed app, particularly for reducing sitting behavior in this population. In light of the small sample size and short study period, further larger-scale evaluation of potential subgroup effects as well as impacts over time for such differently framed apps are warranted.

## Supporting Information

S1 CONSORT Checklist (DOC)

S1 Protocol (PDF)
