# Correction: Effects of Three Motivationally Targeted Mobile Device Applications on Initial Physical Activity and Sedentary Behavior Change in Midlife and Older Adults: A Randomized Trial

**DOI:** 10.1371/journal.pone.0160113

**Published:** 2016-07-21

**Authors:** The PLOS ONE Staff

An incorrect image appears in Fig 2. Please view the correct Fig 2 here. The publisher apologizes for the error.

**Fig 2:**
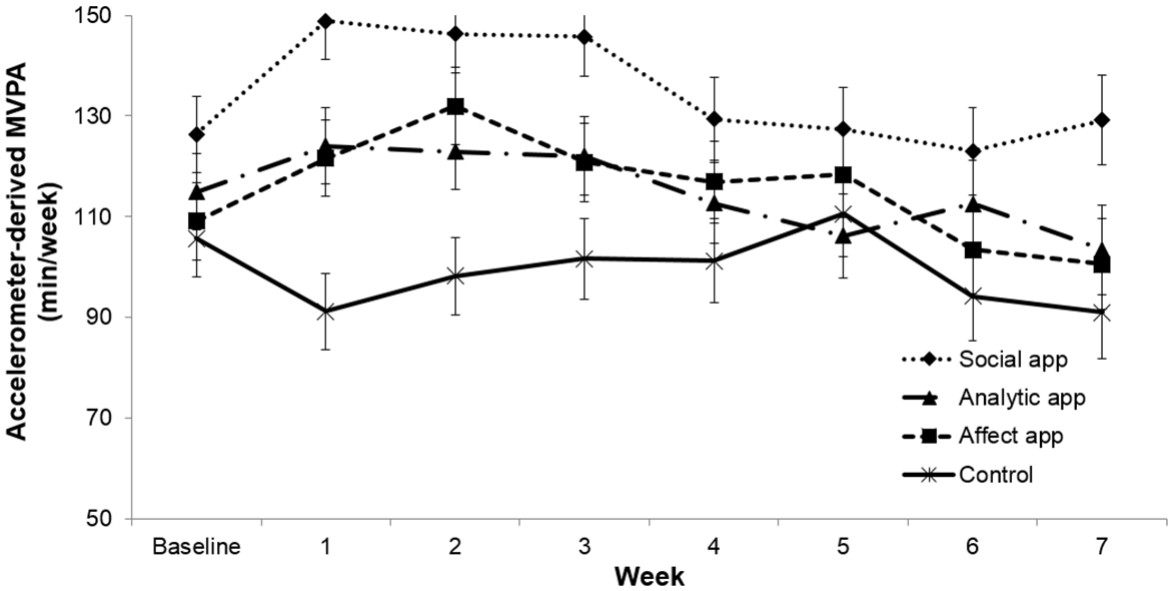
Changes in Accelerometer-Derived MVPA by Study Arm.
