# Supplementary material for: Effects of Three Motivationally Targeted Mobile Device Applications on Initial Physical Activity and Sedentary Behavior Change in Midlife and Older Adults: A Randomized Trial
Source: PLoS One. 2016 Jun 28;11(6):e0156370. doi: 10.1371/journal.pone.0156370 (PMC4924838; doi:10.1371/journal.pone.0156370)
Supplement: S1 Protocol — (PDF) [file pone.0156370.s002.pdf]

Title: Promoting Healthy Lifestyles Using Mobile Phones
Approval Period: 07/02/2015 - 06/30/2016

Continuing Review Form

1. Participant Enrollment

a. Number of participants entered (or number of specimens examined or charts reviewed) since the beginning of study. If this is a combined VA-Stanford study, in addition indicate how many of the participants (or number of VA specimens examined or VA charts reviewed) enrolled with a VA consent. If this is a multi-site study, in addition to the number of participants enrolled locally, include the number of participants enrolled study-wide.

No new participants have been enrolled into this study since the last IRB continuing Review Form was submitted in 2014.

As of June May 21 2014 there were 119 participants in the efficacy pilot study [104 reported last year] [Target enrollment: Up to 280 participants with ~30 for focus groups, 50 for user testing, and up to 200 for the efficacy pilot study.]

In addition, 7 people participated in the focus groups (in 2010) and 4 people participated in the user testing (in 2011)

Total number of participants = 130

b. Number of males, # of females.

Efficacy pilot study:
Males = 30
Females = 89

User testing:
Males = 1
Females = 3

Focus groups :
Males = 1
Females = 6

c. Minority status of participants entered since beginning of study.

Efficacy pilot study:
White/Caucasian = 67
Hispanic/Latino = 25
Black/African American = 0
American Indian/Alaska Native = 0
Asian/Asian American = 12
Native Hawaiian/Pacific Islander = 0
Other = 0

User testing:
White/Caucasian = 7

Focus groups:
White/Caucasian = 4

d. Number of children (less then 18 years) entered since beginning of study.

None

e. Number of other potentially vulnerable subjects (if applicable) entered since the beginning of study, including prisoners, pregnant women, economically and educationally disadvantaged, decisionally impaired and homeless people.

Some of the Latinos enrolled in this study may have been economically or educationally disadvantaged. All members of the research team have been trained to protect the rights of all human subjects in this study.

2. Study Problems/Complications

a. Number of withdrawals of participants from the research (both participant and investigator initiated) since the beginning of the research study. Provide reasons for the withdrawals.

12 participants withdrew. Reasons for withdrawal were:

1 participant lost a study phone and opted not to continue in the study
1 participant was unable to operate the device and opted not to continue in the study
2 participants didn't like the app to which they were assigned.
1 participant had to travel outside the USA which was beyond the range of the Smartphone service provider and servers
1 participant changed jobs and was no longer able to carry the study phone at all times
1 participant had a death in the family and did not wish to continue participating in the study
1 participant had 2 jobs and realized subsequent to enrolling in the study that she didn't have time to be more physically active so opted not to continue in the study.
4 participants gave informed consent to participate in the study and completed a baseline questionnaire but then decided that they didn't want to learn how to use the Smartphone or the app and so withdrew prior to being randomized

b. Number of participants lost to follow-up since the beginning of the study.

None

c. Provide a narrative summary of the adverse events since the last renewal. Indicate whether adverse events experienced by participants are different from those originally anticipated.

None

d. Provide a narrative summary (not a list) of the unanticipated problems involving risks to participants or others that have occurred in the research in the past year. Confirm that all events and information that require prompt reporting to the IRB /research/documents/Events-Info-Report-to-IRB_GUI03P13.pdf (guidance GUI-P13) have been reported as required.

None

e. Provide a narrative summary of all relevant reports received in the past year whether or not the report has been previously submitted to the IRB. Summarize adverse event reports, audit results, and any other reports. Include corrective actions taken as a result of any audits.

None

f. Complaints about the research in the past year.

None

g. Noncompliance: Has there been any agency, institutional, or other inquiry into noncompliance in the study, or any finding of noncompliance concerning a member of the research team? If yes, please explain.

None

3.Study Assessment

a. Provide a narrative summary of any interim findings from your data in the past year.

Preliminary findings indicate that the social app was effective at decreasing sitting time and increasing moderate to vigorous physical activity compared to either the control group or the cognitive app or the affect app.

b. Provide a narrative summary of any recent relevant literature.

Sports Medicine May 2014, Volume 44, Issue 5, pp 671-686
Measuring and Influencing Physical Activity with Smartphone Technology: A Systematic Review

Judit Bort-Roig, Nicholas D. Gilson, Anna Puig-Ribera, Ruth S. Contreras, Stewart G. Trost

In a recent systematic review of the evidence on smartphones and their viability for measuring and influencing physical activity, the authors reviewed 26 articles and found that all studies were conducted in highly economically advantaged countries; 12 articles focused on special populations (e.g. obese patients). Studies measured physical activity using native mobile features, and/or an external device linked to an application. Measurement accuracy ranged from 52 to 100 % (n = 10 studies). A total of 17 articles implemented and evaluated an intervention. Smartphone strategies to influence physical activity tended to be ad hoc, rather than theory-based approaches; physical activity profiles, goal setting, real-time feedback, social support networking, and online expert consultation were identified as the most useful strategies to encourage physical activity change. Only five studies assessed physical activity intervention effects; all used step counts as the outcome measure. Four studies (three pre–post and one comparative) reported physical activity increases (12–42 participants, 800–1,104 steps/day, 2 weeks–6 months), and one case-control study reported physical activity maintenance (n = 200 participants; >10,000 steps/day) over 3 months. The authors conclude that Smartphone use is a relatively new field of study in physical activity research, and consequently the evidence base is emerging. Few studies identified in this review considered the validity of phone-based assessment of physical activity. Those that did report on measurement properties found average-to-excellent levels of accuracy for different behaviors. The range of novel and engaging intervention strategies used by smartphones, and user perceptions on their usefulness and viability, highlights the potential such technology has for physical activity promotion. However, intervention effects reported

c. Attach Data Safety Monitoring Reports in section 16 received in the past year which have not previously been submitted to the IRB.

None

d. Provide a narrative summary of benefits experienced by participants in the past year.

Title: Promoting Healthy Lifestyles Using Mobile Phones
Approval Period: 07/02/2015 - 06/30/2016

Over the past year there have been no participants enrolled in this study.

e. Provide an assessment of whether the relationship of risks to potential benefits has changed.

No change

4. Description of remainder of project:

a. N **Is the study open to enrollment?**

b. Y **Is the study permanently closed to enrollment of new participants?**

c. Y **Have all participants completed all research-related interventions?**

d. N **Are you still engaged in research-related intervention(s)? If yes, please describe.**

e. N **Do you wish to renew this study only for long term follow-up?**

f. Y **Are you only doing data analysis?**

5. Potential Conflict of Interest

N **Is there a change in the conflicting interest status of this protocol?**

6. Protocol Changes

Please note that if these changes involve changes to Radiation Safety or Biosafety, the IRB will hold its approval until Radiation Safety or Biosafety forwards its approval to the IRB.

- **Summarize all of the proposed changes to the protocol application including consent form changes.**

  No changes have been made to the protocol

- **Indicate Level of Risk**

  No Change

- **Describe any other changes.**

  No changes

Protocol Director

Name	Degree (program/year if student)	Title
Abby C King	Ph.D.,	Professor

Title: Promoting Healthy Lifestyles Using Mobile Phones
Approval Period: 07/02/2015 - 06/30/2016

Dept	Mail Code	Phone	Fax	E-mail
Health Research and Policy - Epidemiology	5736	(650) 723-6254	(650) 725-6951	abby.king@stanford.edu
**CITI Training current**				Y

Admin Contact

Name	Degree (program/year if student)	Title
Alexis Katrece Fields	MPH	Research Administrator

Dept	Mail Code	Phone	Fax	E-mail
Medicine - Med/Stanford Prevention Research Center	5541	650-725-3080	(650) 725-6906	Alexis.Fields@stanford.edu
**CITI Training current**				Y

Co-Protocol Director

Name	Degree (program/year if student)	Title
Sandra Jane Winter	PhD	Postdoctoral Fellow

Dept	Mail Code	Phone	Fax	E-mail
Medicine - Med/Stanford Prevention Research Center	5559	(650) 723-4656		sjwinter@stanford.edu
**CITI Training current**				Y

Other Contact

Name	Degree (program/year if student)	Title
Lisa Rosas	PhD, MPH	Instructor of Medicine (Stanford Prevention Research Center)

Dept	Mail Code	Phone	Fax	E-mail
Medicine - Med/Stanford Prevention Research Center	5541	(650) 575-9519	(650) 725-6906	lgrosas@stanford.edu
**CITI Training current**				Y

Faculty Sponsor

Name	Degree (program/year if student)	Title

Dept	Mail Code	Phone	Fax	E-mail

Title: Promoting Healthy Lifestyles Using Mobile Phones
Approval Period: 07/02/2015 - 06/30/2016

**CITI Training current**

Other Personnel

**Name**		**Degree (program/year if student)**		**Title**
Priscilla Padilla		MPH, MPA		
**Dept**	**Mail Code**	**Phone**	**Fax**	**E-mail**
Med/BMIR				ppadilla@stanford.edu
**CITI Training current**				Y

Participant Population(s) Checklist — Yes/No

Population	Yes/No
Children (under 18)	N
Pregnant Women and Fetuses	N
Neonates (0 - 28 days)	N
Abortuses	N
Impaired Decision Making Capacity	N
Cancer Subjects	N
Laboratory Personnel	N
Healthy Volunteers	Y
Students	N
Employees	N
Prisoners	N
Other (i.e., any population that is not specified above)	N

Study Location(s) Checklist — Yes/No

Location	Yes/No
Stanford University	Y
Clinical & Translational Research Unit (CTRU)	
Stanford Hospital and Clinics	
Lucile Packard Children's Hospital (LPCH)	
VAPAHCS (Specify PI at VA)	
Other (Click ADD to specify details)	

General Checklist

Multi-site — **Yes/No**

Title: Promoting Healthy Lifestyles Using Mobile Phones
Approval Period: 07/02/2015 - 06/30/2016

• Is this a multi-site study? A multi-site study is generally a study that involves one or more medical or research institutions in which one site takes a lead role.(e.g., multi-site clinical trial)	N

**Collaborating Institution(s)**	**Yes/No**
• Are there any collaborating institution(s)? A collaborating institution is generally an institution that collaborates equally on a research endeavor with one or more institutions.	N

**Cancer Institute**	**Yes/No**
• Cancer-Related Studies (studies with cancer endpoints), Cancer Subjects (e.g., clinical trials, behavior/prevention) or Cancer Specimens (e.g., blood, tissue, cells, body fluids with a scientific hypothesis stated in the protocol).	N

**Drug /Device**	**Yes/No**
• Investigational drugs, biologics, reagents, or chemicals?	N
• Commercially available drugs, reagents, or other chemicals administered to subjects (even if they are not being studied)?	N
• Investigational Device / Commercial Device used off-label?	N
• IDE Exempt Device (Commercial Device used according to label, Investigational In Vitro Device or Assay, or Consumer Preference/Modifications/Combinations of Approved Devices)	N
• Protocol involves studying potentially addicting drugs?	N

**Clinical Trials**	**Yes/No**
• Click "yes" to confirm that you have accessed the website and read the clinicaltrials.gov reporting requirements provided.	
• This study will be registered on clinicaltrials.gov?	

**Tissues and Specimens**	**Yes/No**
• Human blood, cells, tissues, or body fluids (tissues)?	N
• Tissues to be stored for future research projects?	N
• Tissues to be sent out of this institution as part of a research agreement? For guidelines, please see http://stanford.edu/group/ICO/researcher/reMTA.html	N

http://stanford.edu/group/ICO/researcher/reMTA.html

**Biosafety (APB)**	**Yes/No**
• Are you submitting a Human Gene Transfer investigation using biological agent or recombinant DNA vector? If yes, please complete and attach the Gene Transfer Protocol Application Supplemental Questions to section 16 of the eProtocol application.	N
• Are you submitting a Human study using biohazardous/infectious agents? If yes, refer to the http://www.stanford.edu/dept/EHS/prod/researchlab/bio/index.html Administrative Panel on BioSafety website prior to performing studies.	N
• Are you submitting a Human study using samples from subjects that contain biohazardous/infectious agents? If yes, refer to the https://ehsappprd1.stanford.edu/eprobio/ Administrative Panel on BioSafety website prior to performing studies.	N

**Human Embryos or Stem Cells**	**Yes/No**
• Human Embryos or gametes?	N
• Human Stem Cells (including hESC, iPSC, cancer stem cells, progenitor cells).	N

**Veterans Affairs (VA)**	**Yes/No**
• The research recruits participants at the Veterans Affairs Palo Alto Health Care System(VAPAHCS).	N
• The research involves the use of VAPAHCS non-public information to identify or contact human research participants or prospective subjects or to use such data for research purposes.	N
• The research is sponsored (i.e., funded) by VAPAHCS.	N
• The research is conducted by or under the direction of any employee or agent of VAPAHCS (full- time, part-time, intermittent, consultant, without compensation (WOC), on-station fee-basis, on- station contract, or on-station sharing agreement basis) in connection with her/his VAPAHCS responsibilities.	N
• The research is conducted using any property or facility of VAPAHCS.	N

**Equipment**	**Yes/No**
• Use of Patient related equipment? If Yes, equipment must meet the standards established by Hospital Instrumentation and Electrical Safety Committee (650-725-5000)	N
• Medical equipment used for human patients/subjects also used on animals?	N
• Radioisotopes/radiation-producing machines, even if standard of care? http://www.stanford.edu/dept/EHS/prod/researchlab/radlaser/Human_use_guide.pdf More	N

Title: Promoting Healthy Lifestyles Using Mobile Phones
Approval Period: 07/02/2015 - 06/30/2016

Info

**Payment**	**Yes/No**
• Subjects will be paid/reimbursed for participation? See payment considerations.	N

**Funding**	**Yes/No**
• Training Grant?	N
• Program Project Grant?	N
• Federally Sponsored Project?	Y
• Industry Sponsored Clinical Trial?	N

Funding

Funding - Grants/Contracts

**Funding Administered By:**	STANFORD	**SPO # (if available):**	
**Grant # (if available):**	P30 PAG024957	**Funded By (include pending):**	Stanford Center for Advanced Decision Making in Aging Seed Grant (funded by NIH)

Principal Investigator:

Grant/Contract Title if different from Protocol Title:

Y For Federal projects, are contents of this protocol the same as described in Federal proposal application?
N Is this a Multiple Project Protocol (MPP)?
N Is this protocol under a MPP?

Funding - Fellowships

Gift Funding

Dept. Funding

Other Funding

Expedited Form

A protocol must be no more than minimal risk (i.e., "not greater than those ordinarily encountered in daily life") AND must only involve human subjects in one or more of the following paragraphs.

Select one or more of the following paragraphs:

1. N **Clinical studies of drugs and medical devices only when condition (a) or (b) is met.**
- a) Research on drugs for which an investigational new drug application (21 CFR Part 312) is not required. (Note: Research on marketed drugs that significantly increases the risks or decreases the acceptability of the risks associated with the use of the product is not eligible for expedited review.)
- b) Research on medical devices for which
  - i) an investigational device exemption application (21 CFR Part 812) is not required; or
  - ii) the medical device is cleared/approved for marketing and the medical device is being used in accordance with its cleared/approved labeling.

2. N **Collection of blood samples by finger stick, heel stick, ear stick, or venipuncture as follows:**
- a) from healthy, nonpregnant adults who weigh at least 110 pounds. For these subjects, the amounts drawn may not exceed 550 ml in an 8 week period and collection may not occur more frequently than 2 times per week; or
- b) from other adults and children, considering the age, weight, and health of the subjects, the collection procedure, the amount of blood to be collected, and the frequency with which it will be collected. For these subjects, the amount drawn may not exceed the lesser of 50 ml or 3 ml per kg in an 8 week period and collection may not occur more frequently than 2 times per week.

3. N **Prospective collection of biological specimens for research purposes by non invasive means.**

4. Y **Collection of data through non invasive procedures (not involving general anesthesia or sedation) routinely employed in clinical practice, excluding procedures involving x-rays or microwaves. Where medical devices are employed, they must be cleared/approved for marketing. (Studies intended to evaluate the safety and effectiveness of the medical device are not generally eligible for expedited review, including studies of cleared medical devices for new indications.)**

Examples:
- a) physical sensors that are applied either to the surface of the body or at a distance and do not involve input of significant amounts of energy into the subject or an invasion of the subject's privacy;
- b) weighing or testing sensory acuity;
- c) magnetic resonance imaging;
- d) electrocardiography, electroencephalography, thermography, detection of naturally occurring radioactivity, electroretinography, ultrasound, diagnostic infrared imaging, doppler blood flow, and echocardiography;
- e) moderate exercise, muscular strength testing, body composition assessment, and flexibility testing where appropriate given the age, weight, and health of the individual.

5. N **Research involving materials (data, documents, records, or specimens) that have been collected, or will be collected solely for nonresearch purposes (such as medical treatment or diagnosis). (NOTE: Some research in this paragraph may be exempt from the HHS regulations for the protection of human subjects. 45 CFR 46.101(b)(4). This listing refers only to research that is not exempt.)**

6. Y **Collection of data from voice, video, digital, or image recordings made for research**

purposes.

7. Y **Research on individual or group characteristics or behavior(including, but not limited to, research on perception, cognition, motivation, identity, language, communication, cultural beliefs or practices, and social behavior) or research employing survey, interview, oral history, focus group, program evaluation, human factors evaluation, or quality assurance methodologies. (NOTE: Some research in this category may be exempt from the HHS regulations for the protection of human subjects. 45 CFR 46.101(b)(2) and (b)(3). This listing refers only to research that is not exempt.)**

Resources:

a) **Qualified staff.**

Please state and justify the number and qualifications of your study staff.

Abby King, Ph.D. - Protocol Director
Extensive experience leading clinical trials of physical activity adoption interventions using state-of-the-art technology (e.g., automated phone systems, pdas).

Lisa Goldman Rosas, Ph.D. - Research Scientist.
Fluent in English and Spanish. Extensive experience working in Latino communities directing clinical trials and all associated aspects such as recruitment, participant retention, intervention delivery and outcomes evaluation.

Sandra J Winter, Ph.D. - Project Manager
Experience managing and coordinating clinical trials of physical activity adoption interventions among older adults including: recruitment, participant retention, intervention delivery and outcomes evaluation.

b) **Training.**

Describe the training you will provide to ensure that all persons assisting with the research are informed about the protocol and their research-related duties and functions.

The research team meets weekly to address ongoing research activities in the area of physical activity adoption and intervention implementation. This particular project will be discussed during the weekly lab meeting throughout its development and implementation to monitor progress and assign responsibilities. This project will also be discussed during the weekly "intervention" meeting to discuss any specific intervention implementation and training issues as well as during the weekly "assessment" meeting to address any measurement or assessment issues. Additional support will be provided, as needed.

c) **Facilities.**

Please describe and justify.

The Stanford Prevention Research Center has extensive research facilities with the computer, statistical software, telephone and office space needed for this particular project.

d) **Sufficient time.**

Explain whether you will have sufficient time to conduct and complete the research. Include how much time is required.

The current project is anticipated to take 2 years to complete. Approximately 9 months for development, 10 months for intervention pilot testing, and 5 months for Data compilation, cleaning, analysis, and report writing.

e) Access to target population.

Explain and justify whether you will have access to a population that will allow recruitment of the required number of participants.

We are recruiting from the general population, and have previous experience using a variety of recruitment methods including newspaper ads and attending community events that have successfully resulted in adequate enrollment.

f) Access to resources if needed as a consequence of the research.

State whether you have medical or psychological resources available that participants might require as a consequence of the research when applicable. Please describe these resources.

The likelihood of participants needing medical or psychological resources as a result of participating in this project is very low. However, we will ensure that participants have a primary care provider that they can access, if in need. In addition, the project director is a clinical psychologist and can provide appropriate referral and resource information pertaining to mental health.

g) Lead Investigator or Coordinating Institution in Multi-site Study.

Please explain (i) your role in coordinating the studies, (ii) procedures for routine communication with other sites, (iii) documentation of routine communications with other sites, (iv) planned management of communication of adverse outcomes, unexpected problems involving risk to participants or others, protocol modifications or interim findings.

1. Purpose

a) In layperson's language state the purpose of the study in 3-5 sentences.

The purpose of this research is to test a program to increase physical activity and reduce sedentary behavior using motivational messages over a cell phone.

b) State what the Investigator(s) hope to learn from the study. Include an assessment of the importance of this new knowledge.

We want to learn if a behavioral intervention for promoting increased physical activity and decreased sedentary behavior via state-of-the-art mobile phones will be efficacious at improving these behaviors relative to commercially available Android applications as a control. If efficacious, this type of intervention program could be disseminated to a wide variety of sedentary and underactive adults at a relatively low cost. This could have a large impact on promoting improved health such as reduced obesity, a key issue problem within the U.S.

c) Explain why human subjects must be used for this project. (i.e. purpose of study is to test efficacy of investigational device in individuals with specific condition; purpose of study is to examine specific

Title: Promoting Healthy Lifestyles Using Mobile Phones
Approval Period: 07/02/2015 - 06/30/2016

behavioral traits in humans in classroom or other environment)

Human subjects are necessary to examine the efficacy of an intervention for promoting increased physical activity and decreased sedentary behaviors among adults.

2. Study Procedures

a) Please SUMMARIZE the research procedures, screening through closeout, which the human subject will undergo. Refer to sections in the protocol attached in section 16, BUT do not copy the clinical protocol. Be clear on what is to be done for research and what is part of standard of care.

Screening procedures:
Participants in both the focus group and intervention study will be
screened using one of two methods, either the attached telephone screen scripts/forms or
the
online screening to ensure
participants meet eligibility requirements for each phase. The
telephone/online screens are expected to take approximately 10 minutes. Participants in
the
user testing phase will self-select to be a part of the study and thus will not be screened
prior
to receiving the application.

There will be three phases to this research, a focus group/development stage, a user
testing
phase, and an intervention phase.

Focus Group/Software Development
Approximately 30 participants will be asked to handle a mobile phone and give feedback
about their experiences with it (e.g., the way it looks, how it interacts), to improve upon
the
overall usability of the applications being developed. These participants responses during
the
focus groups will not be linked to individual characteristics. Participants will be asked to
participate for up to 8 hours in focus group meetings.

User Testing Phase:
Participants for the user testing phase would be recruited from the
Android Market. It is estimated that approximately 50 participants
will be recruited for this phase of the study.

The User Testing Informed Consent Statement would be displayed on the
phone for individuals to read and agree or decline participation in
the study. If participants consent to participate, minimal
demographic (i.e., age, gender, ethnicity) and other information (i.e, height, weight,
physical
activity level, readiness to be physically active) will be assessed. No other PHI will be
gathered
for user testing. Participants will be excluded from the user testing phase if they are: a)
under

the age of 18; or b) not ready to be regularly physically active.

Following eligibility determination, participants will be randomized to receive one of three mobile phone-based applications including: a) a feedback only (i.e., the mobile phone monitors an individuals physical activity level and provides feedback passively on the mobile phone's home screen). The mobile phone will display the user's physical activity as a growing garden; b) feedback+anchor (i.e., similar to the previous application but with the addition of a line to help determine when recommendations for physical activity are met).

The mobile phone will display the user's physical activity as a growing garden, and a progress bar to display their progress toward reaching physical activity recommendations; c) feedback+anchor+scheduling (i.e., same as b but with a goal-setting/scheduling component). The mobile phone will display the user's physical activity as a growing garden, and a progress bar to display their progress toward reaching physical activity recommendations, and prompts to encourage them to set physical activity goals and schedule times for physical activity on their calendar. Participants will be allowed to use the application for up to 4 months.

Data about our participants physical activity levels will be gathered while participants use the application and transferred back to us on an encrypted channel and stored on a password-protected server. In addition, participants will be asked to anonymously fill out surveys describing their experiences with using the applications. Participants will have the opportunity to drop out of the user testing at any point by uninstalling the application.

Intervention/efficacy pilot study Phase:
Up to 120 participants (30 per arm) will be recruited for this phase of the study. Participants will be screened using the technique described above. If eligible, they will be invited to an orientation session to learn more about the project. If participants are still interested in the project after the orientation session, informed consent will be received from all participants in the intervention phase and participants will either be given a new "smart phone" loaded with software to monitor their physical activity and sedentary behavior or, if they own an appropriate Android smart phone, we will randomize participants and install the applications on their phones. Participants will be monitored for one week via the mobile phones. Participants will be randomized to one of four conditions: a) the cognitive application (i.e., goal-setting, problem-solving, feedback); b) the affect application (i.e., operant conditioning, which is positively reinforcing a behavior when it occurs, with feedback delivered via a bird avatar); c) the social application (i.e., social comparison of activity, a message board, and

an
opt-in component to share progress with family and friends); or d)a free commercially available Android application (i.e., Calorific or Stress Relief Tips, with participants given the option of choosing). The following week, participants will return to Stanford to complete baseline questionnaires including interviews about physical activity over the past 7 days and
smart phone acceptability. Participants will then receive an intervention that is specific to the
assigned condition and participants will be followed for up to 4 months.

Participants will also complete daily self-report information about their beliefs and behaviors using the mobile phone. At the end of up to four months, participants will come to Stanford for a debriefing session. At that time,
the smart phone will be retrieved from the participants who were given smart phones and participants will be asked to complete questionnaires and be interviewed about physical activity during the past seven days
and general program satisfaction. Participants will also be encouraged to discuss any potential problems that arose during the intervention with the investigators directly during this debriefing session. For those participants randomized to the "social" application, participants will be given the debriefing statement and research personnel will follow the debriefing script for describing the use of confederates during the course of the study with regard to establishing social comparisons and the message board.

In addition to using the SmartPhones to promote improvements to health behaviors, participants will be offered the opportunity of wearing a SenseCam during waking hours for
up to 5 days during the study period. The SenseCam is a camera that is worn around the neck and that automatically takes pictures of the environment. The camera is fitted with a temperature sensor, a light color and intensity sensor, a passive infra-red motion detector, a
multi-axis accelerometer and a compass. Changes in sensor readings automatically trigger the camera to take up to 12 pictures per minute. The SenseCam has a privacy button which allows users to turn the camera off and back on at any time.

b) Explain how the above research procedures are the least risky that can be performed consistent with sound research design.

Minimal risk is achieved through careful screening for healthy volunteers, counseling in the safe adoption of increased physical activity and decreased sedentary behaviors, active monitoring of participants, and providing the debriefing statement at the completion of the study. An intervention period of up to 4 months is the minimum necessary to adequately test for differences between the intervention arms. We plan to include up to 3 confederates in each of the two teams that research participants will be members of in the social app. Specifically, we will Establish a group level of activity that is meant to establish appropriate social norms thought to maximize motivation for change within the group. In addition, research personnel will respond as these "confederates" within the social app message board to help stimulate conversation. This type of confederate procedure is common in other psychological research and is required for the manipulation to work effectively. Although there is deception, the deception does not pass beyond the minimal risk

threshold required for the use of deception, particularly because we will provide full disclosure of the deception immediately after the completion of the study via the debriefing statement and script. Note that the debriefing will be conducted immediately after the completion of the study by research personnel who are well-informed about the research project and the deception. Finally, for the participants in the social arm of the study, participants will be given the option of opting into a "share" component of the application, whereby successes in their activity levels (e.g., walking enough to meet physical activity guidelines) will be automatically shared with a self-selected group of family and friends via text message and/or email. This component will only be available as an opt-in, however, and thus nothing about the person's activity level will be shared with family and friends without their explicit inclusion of the family and friends within the share data set. Further, participants will have the option of removing family and friends from this component of the application at any time.

c) State if deception will be used. If so, provide the rationale and describe debriefing procedures. Since you will not be fully informing the participant in your consent process and form, complete an alteration of consent (in section 13). Submit a debriefing script (in section 16).

With regard to the social application, we will be using confederates in the social application. This is being done for three reasons: 1) For the activity comparison component, previous research suggests the power of social norms to influence behaviors, if they are set slightly higher but achievable goals. With confederates, we can ensure that an appropriately motivational social norm that is in line with national guidelines for physical activity (i.e., at least 150 minutes per week of moderate intensity physical activity) and sedentary behavior (i.e., 8 or less hours of sedentary behavior per day) is established. Both of these milestones will be higher for physical activity and lower for sedentary behavior than our participants baseline level of activity but achievable based on prior experience working with mid-life and older adults who have achieved these goals throughout an intervention trial. This manipulation is vital to ensure the proper test of our social application. 2) As we will be utilizing a rolling recruitment method, the first participants will not have any other group members. With confederates, we can ensure that all participants, including the first few participants, will receive an "active" intervention. 3) Although some participants may be interested in reading message board postings, they may not be as interested in also posting messages. By allowing research personnel to pose as confederates within the message board, we will spur conversation and offer appropriate content for the message board regardless of our participants' personal engagement with posting to the message board. This is particularly important for the small sample size of participants using the social application.

To rectify the above problems, we plan to include up to 4 confederates in each of the two teams in the social app. Specifically, we will use a database of activity levels that are meant to establish appropriate social norms for maximizing motivation for change within the group and displaying that information as the activity level of fellow team members participating in the social arm. In addition, research personnel will respond as these "confederates" within the social app message board to stimulate conversation.

This type of confederate procedure is common in other psychological research and is required for the manipulation to work effectively. Although there is deception, the deception does not pass beyond the minimal risk threshold required for the use of deception, particularly because we will provide full disclosure of the deception immediately after the completion of the study via the debriefing statement and script.

The debriefing will be conducted immediately after the completion of the study and will be provided by research personnel who are well-informed about the research project and the deception. Research personnel will follow the debriefing script and also provide the debriefing statement to all participants in the social application.

d) State if audio or video recording will occur. Describe what will become of the recording after use, e.g., shown at scientific meetings, erased. Describe the final disposition of the recordings.

Low resolution images taken by the SenseCam will be treated in the same secure manner as other data gathered from participants in terms of use, storage and final disposition.

e) Describe alternative procedures or courses of treatment, if any, that might be advantageous to the participant. Describe potential risks and benefits associated with these. Any standard treatment that is being withheld must be disclosed in the consent process and form. (i.e. standard-of-care drug, different interventional procedure, no procedure or treatment, palliative care, other research studies).

None. The alternative to this study is not to participate and to engage in increasing physical activity or decreasing sedentary independently.

f) Will it be possible to continue the more (most) appropriate therapy for the participant(s) after the conclusion of the study?

N/A

g) Study Endpoint. What are the guidelines or end points by which you can evaluate the different treatments (i.e. study drug, device, procedure) during the study? If one proves to be clearly more effective than another (or others) during the course of a study, will the study be terminated before the projected total participant population has been enrolled? When will the study end if no important differences are detected?

Each participant in the intervention phase will participate for up to four months. Interventions will not be terminated early.

Participants in the user testing phase will be allowed to discontinue participation at any time but have the opportunity to use the application for up to 4 months (or intended length of use in the intervention trial).

3. Background

a) Describe past experimental and/or clinical findings leading to the formulation of the study.

The chronic diseases that constitute the major killers of American adults, among them cardiovascular disease, cancer, and other diseases of aging, are inextricably linked with key health behaviors. These include regular physical activity and, more recently, sedentary behaviors such as prolonged television viewing. Regular adherence to healthful behavioral regimens remains poor across the majority of Americans. Recent advances in communication technologies such as 'smart phones' offer a potentially transformative platform for addressing common barriers to adherence through delivering convenient, sustainable, and contextually meaningful adherence strategies.

b) Describe any animal experimentation and findings leading to the formulation of the study.

Not Applicable.

4. Radioisotopes or Radiation Machines

a) List all standard of care procedures using ionizing radiation (radiation dose received by a subject that is considered part of their normal medical care). List all research procedures using ionizing radiation (procedures performed due to participation in this study that is not considered part of their normal medical care). List each potential procedure in the sequence that it would normally occur during the entire study. http://www.stanford.edu/dept/EHS/prod/researchlab/radlaser/Human_use_guide.pdf More Info

Identify Week/Month of study	Name of Exam	Identify if SOC or Research

b) For research radioisotope projects, provide the following radiation-related information:

Identify the radionuclide (s) and chemical form (s).

For the typical subject, provide the total number of times the radioisotope and activity will be administered (mCi) and the route of administration.

If not FDA approved provide dosimetry information and reference the source documents (package insert, MIRD calculation, peer reviewed literature).

c) For research radiation machine projects, provide the following diagnostic procedures:

For well-established radiographic procedures describe the exam.

For the typical subject, identify the total number of times each will be performed on a single research subject.

For each radiographic procedure, provide the setup and technique sufficient to permit research subject dose modeling. The chief technologist can usually provide this information.

For radiographic procedures not well-established, provide FDA status of the machine, and information sufficient to permit research subject dose modeling.

d) For research radiation machine projects, provide the following therapeutic procedures:

For a well-established therapeutic procedure, identify the area treated, dose per fraction and number of fractions. State whether the therapeutic procedure is being performed as a normal part of clinical management for the research participants's medical condition or whether it is being performed because the research participant is participating in this project.

For a therapeutic procedure that is not well-established, provide FDA status of the machine, basis for dosimetry, area treated, dose per fraction and number of fractions.

5. Devices

a) Please list in the table below all Investigational Devices (including Commercial Devices used off-label) to be used on participants

b) **Please list in the table below all IDE Exempt Devices** (**Commercial Device used according to label, Investigational In Vitro Device or Assay, or Consumer Preference/Modifications/Combinations of Approved Devices**) **to be used on participants.**

6. Drugs, Reagents, or Chemicals

a) **Please list in the table below all investigational drugs, reagents or chemicals to be administered to participants.**

b) **Please list in the table below all commercial drugs, reagents or chemicals to be administered to participants.**

7. Medical Equipment for Human Subjects and Laboratory Animals

If medical equipment used for human patients/participants is also used on animals, describe such equipment and disinfection procedures.

N/A

8. Participant Population

a) **State the following:** (**i**) **the number of participants expected to be enrolled at Stanford-affiliated site**(s); (**ii**)**the total number of participants expected to enroll at all sites;** (**iii**) **the type of participants** (**i.e. students, patients with certain cancer, patients with certain cardiac condition**) **and the reasons for using such participants.**

Up to 280 participants will be recruited, with approximately 30 for the focus groups, 50 for the user testing, and up to 200 for the efficacy pilot study. All participants in the focus groups and efficacy pilot study will be recruited at Stanford University. These individuals will be healthy women and men ages 25 and older who meet the eligibility criteria for study participation. These subjects will be recruited from the San Francisco Bay area, CA. Participants in the user testing phase will be recruited from the Android Market, will be men and women ages 18 and older who meet eligibility criteria.

b) **State the age range, gender, and ethnic background of the participant population being recruited.**

Ages 25 and older, men and women, and all ethnic backgrounds in the greater San Francisco Bay Area for the focus group and intervention trial. Participants in the user testing will be ages 18 and older, men and women, and all ethnic backgrounds recruited from the Android Market.

c) **State the number and rationale for involvement of potentially vulnerable subjects in the study (including children, pregnant women, economically and educationally disadvantaged, decisionally impaired, homeless people, employees and students). Specify the measures being taken to minimize the risks and the chance of harm to the potentially vulnerable subjects and the additional safeguards that have been included in the protocol to protect their rights and welfare.**

No potentially vulnerable subjects.

d) **If women, minorities, or children are not included, a clear compelling rationale must be provided (e.g.,**

disease does not occur in children, drug or device would interfere with normal growth and development, etc.).

Women and minorities are included.
Children are not included for two reasons:
1)this is an initial feasibility study of an automated mobile-phone based intervention program to increase physical activity and decrease television viewing among adults.
2)There is extensive research focused on promoting reduced television viewing among children but little research among adults.

e) **State the number, if any, of participants who are laboratory personnel, employees, and/or students. They should render the same written informed consent. If payment is allowed, they should also receive it. Please see Stanford University policy.**

None.

f) **State the number, if any, of participants who are healthy volunteers. Provide rationale for the inclusion of healthy volunteers in this study. Specify any risks to which participants may possibly be exposed. Specify the measures being taken to minimize the risks and the chance of harm to the volunteers and the additional safeguards that have been included in the protocol to protect their rights and welfare.**

All participants in the focus groups and the intervention trial will be sedentary but otherwise healthy volunteers. Participants in the user testing will be healthy adults who report being ready to engage in regular physical activity.

Healthy volunteers are included because we are interested in seeing the utility of this program within a general population.

Psychological risks of the evaluation: There is a remote risk that persons completing questionnaires focusing on behavioral or psychological content may become distressed. There is no evidence that any permanent psychological dysfunction has resulted from such assessments. For participants within the social arm of the study, deception will take place, however, the level of deception is quite minimal as the only deception is the inclusion of confederate data to establish appropriate social norms for physical activity and sedentary behaviors that are in line with national recommendations and thus achievable by all participants. Further, confederates will be used to help establish dialogues within the social arm's message board. To minimize risks from this procedure, all participants in the social app will be debriefed immediately following the completion of the study by trained research personnel.

Medical risks of increased physical activity or reduced sedentary behavior programs: The major risks of physical activity programs by initially inactive overweight persons are orthopedic and cardio-respiratory. Orthopedic problems primarily are of the overuse variety and usually can be treated by rest and change in the mode of physical activity. Frequent minor problems will occur, including temporary soreness or irritation of muscles, tendons and joints. The likelihood of orthopedic and cardio-respiratory risks are greatly minimized through the use of a moderate-intensity exercise program that involves primarily mild to moderate-intensity walking, as
proposed in the current study, and an individualized, progressive approach to physical activity as proposed in the current program.

g) **How will you identify participants for recruitment? (E.g., by: chart review; referral from treating physician; response to ad). Attach recruitment materials in Section #16 (Attachments). All Final or revised recruitment materials, flyers, etc. must be submitted to the IRB for review and approval before use. You may not contact potential participants prior to IRB approval. See Advertisements: Appropriate Language for Recruitment Material.**

The primary recruitment methods for both the focus-group and the intervention study will consist of community-based media promotion supplemented with targeted postings and mailings. In addition, we will direct our participants to our website: http://healthyaging.stanford.edu/miles.html. Recruitment for the user testing will be accomplished via the Android Market.

h) Inclusion and Exclusion Criteria.

Identify inclusion criteria.

1) age 45 and older
2) currently sedentary (less than 60 minutes per week of moderate activity)
3) eat less than 5 servings of fruits and vegetables combined on most days
4) Does not currently own or regularly use a Smartphone
5) willing to be randomly assigned to different conditions
6) Sedentary for more than 7 hours per day.

Identify exclusion criteria.

1) free of clinically evident cardiovascular disease,
2) free of any other medical condition or disorder (e.g., orthopedic disabilities) that would limit participation in moderate intensity physical activities akin to brisk walking

i) Describe your screening procedures, including how qualifying laboratory values will be obtained. If you are collecting personal health information prior to enrollment (e.g., telephone screening), please request a waiver of authorization for recruitment (in section 15).

Potential participants will undergo either a telephone or online screening interview to determine if they meet study eligibility criteria. Participants in the focus-group will be asked a series of questions about their age, current patterns of physical activity, sedentary activity, and cell phone use. Participants will also have a thorough explanation of the study provided to them to help them determine if they are or are not interested in participating.

Participants in the intervention study will be asked a series of questions about their age, current patterns of physical activity, sedentary activity, cell phone use, and current medical conditions. Participants will also have a thorough explanation of the study provided to them to help them determine if they are or are not interested in participating.

j) Describe how you will be cognizant of other protocols in which participants might be enrolled. Please explain if participants will be enrolled in more than one study.

During the telephone screening, participants will be asked if they are currently participating in other research protocols. Participants will be allowed to enroll in the current study if their involvement does not interfere with the other protocols, if the other protocols do not interfere with this study, or if their participation in more than one protocol does not create undue burden for the participant.

k) Payment. Explain the amount and schedule of payment, if any, that will be paid for participation in the study. Substantiate that proposed payments are reasonable and commensurate with the expected contributions of participants and that they do not constitute undue pressure on participants to volunteer for the research study. Include provisions for prorating payment. See payment considerations

Participants will receive a $20 gift card for participating in the study.

l) Costs. Please explain any costs that will be charged to the participant.

There will be no cost associated with participation.

m) Estimate the probable duration of the entire study. Also estimate the total time per participant for: (i) screening of participant; (ii) active participation in study; (iii) analysis of participant data.

It is estimated that the study will last for two years.

Focus group participants will be asked to complete a 10 minute telephone screen and no more than 8 hours of focus group discussion scheduled across a maximum of 4 days.

Intervention group members will be involved in 10 minutes of the telephone/online screen and then will carry the smart phone for up to 4 months. Participants will complete an on-line survey at the beginning and end of the intervention period, each of which takes approximately 45 minutes.

Data analysis will occur during the final 5 months of the project.

9. Risks

a) For the following categories include a scientific estimate of the frequency, severity, and reversibility of potential risks. Wherever possible, include statistical incidence of complications and the mortality rate of proposed procedures. Where there has been insufficient time to accumulate significant data on risk, a statement to this effect should be included. (In describing these risks in the consent form to the participant it is helpful to use comparisons which are meaningful to persons unfamiliar with medical terminology.)

Investigational devices.

none.

Investigational drugs. Information about risks can often be found in the Investigator's brochure.

none.

Commercially available drugs, reagents or chemicals. Information about risks can often be found in the package insert.

none.

Procedures to be performed. Include all investigational, non-investigational and non-invasive procedures (e.g., surgery, blood draws, treadmill tests).

none.

Radioisotopes/radiation-producing machines (e.g., X-rays, CT scans, fluoroscopy) and associated risks.

none.

Physical well-being.

There is a small risk of injury consistent with initiation of a new physical activity program. Such injuries are temporary and minor (i.e., muscle fatigue and soreness). These risks are minimized by encouraging adults to gradually increase the frequency and intensity of physical activity, and engage in proper warm-up and cool-down before and after exercising.

Psychological well-being.

There is a small risk of psychological discomfort when answering questions of a personal nature. These risks are minimized by informing subjects that they do not have to respond to any questions that they do not want to answer, and there are no negative consequences for withholding responses. Further, there is a small risk of psychological discomfort for participants in the social arm of the study based on the deception. This psychological discomfort will be minimized by careful debriefing by trained personnel immediately following the completion of the study.

Economic well-being.

none.

Social well-being.

none.

Overall evaluation of Risk.

Low - innocuous procedures such as phlebotomy, urine or stool collection, no therapeutic agent, or safe therapeutic agent such as the use of an FDA approved drug or device.

b) If you are conducting international research, describe the qualifications/preparations that enable you to both estimate and minimize risks to participants. Also complete the 'http://humansubjects.stanford.edu/research/documents/intl_rsch_APP-11.doc' International Research Form and attach it in the Attachments section. If not applicable, enter N/A.

not applicable.

c) Describe the planned procedures for protecting against and minimizing all potential risks. Include the means for monitoring to detect hazards to the participant (and/or to a potential fetus if applicable). Include steps to minimize risks to the confidentiality of identifiable information.

Participants will be be instructed in the safe, gradual development of increased physical activity that is appropriate to their level of experience, comfort, and convenience. Participants will be encouraged to progress slowly and gradually to minimize physical discomforts. All participants will be instructed to contact the research staff to report any change in their physical or mental health. In addition, during the phone's gathering of daily self-report data, participants will be asked about any potential physical or psychological problems to further improve the likelihood of identifying any problems early. The staff will be prepared to address these concerns and/or provide referrals for medical care in the event of an exercise-related injury (strained muscles, fall, and acute pain in muscles or joints). Due to the minimal risks of harm from moderate physical activity, subjects will not be terminated by investigators.

d) Explain the point at which the experiment will terminate. If appropriate, include the standards for the termination of the participation of the individual participant Also discuss plans for ensuring necessary medical or professional intervention in the event of adverse effects to the participants.

The study will terminate when all subjects have completed the protocol.

10. Benefits

a) Describe the potential benefit(s) to be gained by the participants or by the acquisition of important knowledge which may benefit future participants, etc.

We believe that all participants will benefit by learning (from the study questionnaires) about their health status and physical activity levels. In addition, those in the intervention study who successfully improve their physical activity levels and/or sedentary behaviors will have achieved desired goals. Over the study period all intervention study participants will benefit from receiving health information.

We believe society will benefit from the results of this project in that it will help to inform further research aimed at developing effective and appropriate interventions for promoting increased physical activity and decreased sedentary behaviors among overweight adults. This study will shed light on the best ways to use state-of-the-art mobile phone technology for promoting improved health and wellness.

This technology could conceivably be disseminated relatively quickly and at a low cost relative to more traditional health intervention programs. Therefore, these results could have a significant public health impact within the realm of health promotion.

11. Privacy and Confidentiality

Privacy Protections

a) **Describe how the conditions under which interactions will occur are adequate to protect the privacy interests of participants (e.g., privacy of physical setting for interviews or data collection, protections for follow-up interactions such as telephone, email and mail communications).**

Screening will be conducted on a private phone in a private office for all participants or on a secure website.

Focus groups will be conducted within small groups of no more than 5 participants at a time. Participants will be told of the importance of confidentiality of other participants when participating in research studies (i.e., whatever gets discussed in the group does not leave the group). First names only will be used during discussions to further protect individual group members. Group discussions will be held in private conference rooms at the Stanford Prevention Research Center.

To ensure the privacy of participants within the intervention phase, participants will be seen in a private office to collect all baseline and follow-up data and to be taught the basics on the use of the cell phone software. All data gathered automatically will be sent over a secure encrypted telephone network to a password protected server.

Confidentiality Protections

b) **Specify PHI (Protected Health Information). PHI is health information linked to HIPAA identifiers (see above). List BOTH health information AND HIPAA identifiers. If you are using STRIDE, use the Clinical Data Work Sheet to ensure that your request will match your IRB-approved protocol. Be consistent with information entered in section 15a.**

PHI collected will include: name, ethnicity, date of birth, health history, street address, zip code, email address, and telephone number and current medical conditions and medications. PHI will be use (1) to determine eligibility for the study and (2) to contact participants to schedule study activities.

c) **You are required to comply with University Policy that states that ALL electronic devices: computers (laptops and desktops; OFFICE or HOME); smart phones; tablets; external hard disks, USB drives, etc. that may hold identifiable participant data will be password protected, backed up, and encrypted. See http://med.stanford.edu/datasecurity/ for more information on the Data Security Policy and links to encrypt your devices.**

Provide any additional information on ALL data security measures you are taking. You must use secure databases such as RedCap https://clinicalinformatics.stanford.edu/services/redcap.html. If you are unsure of the security of the system, check with your Department IT representative. Please see

http://med.stanford.edu/irt/security/ for more information on IRT Information Security Services and http://www.stanford.edu/group/security/securecomputing/mobile_devices.html for more information for securing mobile computing devices. Additionally, any PHI data on paper must be secured in an locked environment.

By checking this box, You affirm the aforementioned. Y

Data will be maintained electronically and on paper. Electronic files will be encrypted and stored on University servers that have firewalls in place and are dependent upon passwords for access. All computers used for this study will be password protected. Mobile devices will be equipped with a personal firewall, and software to enable full disk and/or folder or file encryption. Decryption keys will be locked in a safe location. Paper files will be stored in locked file cabinets in locked offices.

d) Describe how data or specimens will be labeled (e.g. name, medical record number, study number, linked coding system) or de-identified. If you are de-identifying data or specimens, who will be responsible for the de-identification? If x-rays or other digital images are used, explain how and by whom the images will be de-identified.

Data other than demographic information will not use names as an identifier. A randomly assigned research ID number will be used.

e) Indicate who will have access to the data or specimens (e.g., research team, sponsors, consultants) and describe levels of access control (e.g., restricted access for certain persons or groups, access to linked data or specimens).

Members of the research team will have access to data. Such access will be password-protected (via computer) and only staff will have access to keys for locked files. Staff involved in interviewing/screening will only have access to data in the collection phase; staff involved in the data analysis phase will not have access to data with original identification.

f) If data or specimens will be coded, describe the method in which they will be coded so that study participants' identities cannot be readily ascertained from the code.

Participants will receive a randomly assigned research ID number. A "key" will be maintained which links the participants to their previous identification. The key will be maintained
electronically (with regularly scheduled data backups), using data-encryption procedures and stored on University maintained servers with electronic firewall protection. "Strong" computer passwords will be required to gain access to the key; passwords will be changed routinely.

g) If data or specimens will be coded, indicate who will maintain the key to the code and describe how it will be protected against unauthorized access.

The PI and other senior level staff will have access to the key (maintained electronically). This data will be password protected. Internal access will be strictly limited on a 'need to know' basis.

h) If you will be sharing data with others, describe how data will be transferred (e.g., courier, mail) or transmitted (e.g., file transfer software, file sharing, email). If transmitted via electronic networks, describe how you will secure the data while in transit.See http://www.stanford.edu/group/security/securecomputing/. http://www.stanford.edu/group/security/securecomputing/. Additionally, if you will be using or sharing

PHI see http://hipaa.stanford.edu/policy_security.html http://hipaa.stanford.edu/policy_security.html.

If data sharing is required, we will transfer data electronically in accordance with the following guidelines:

o Transmission of data will be encrypted through the use of 128-bit SSL or other industry acceptable methods.
o Wireless communication will be encrypted using Wi-Fi Protected Access (WPA), VPN, or 128-bit SSL

i) **How will you educate research staff to ensure they take appropriate measures to protect the privacy of participants and the confidentiality of data or specimens collected (e.g. conscious of oral and written communications, conducting insurance billing, and maintaining paper and electronic data)?**

The staff orientation meeting will include a discussion of confidentiality and how this is to be maintained throughout the course of the study. All staff will have taken the required online human subjects course on this topic. In addition, this topic will be regularly addressed during weekly staff meetings where staff will be encouraged to ask questions pertaining to this issue.

12. Potential Conflict of Interest

The investigators listed below are required to disclose any financial interests that reasonably appear to be related to this protocol. An email has been sent to them by OPACS (Outside Professional Activities Certification System), and each must respond Yes or No to the financial interest question in the email. If No, they're done. If Yes, they go directly to the OPACS dashboard and file their disclosure. Investigators who do not receive an email from OPACS must go directly to the OPACS dashboard and fill out information there.

You will be unable to submit this protocol until the Financial Interest disclosure is completed by all investigators.

This protocol will not be approved until all investigators who have responded YES to Financial Interests have submitted their OPACS disclosure and OPACS review has been completed by the COI Manager.

Contact https://helpsu.stanford.edu/helpsu/3.0/helpsu-form?pcat=OPACS OPACS HelpSU for any issues with OPACS.

Note: If any changes to disclosures are made while this page is open, simply reload the page to see current information.

Investigators	Role	Email	Has Financial Interest?	Date Financial Interest Answered	Date OPACS Disclosure Submitted	Date OPACS Review Completed
Abby C King	PD	abby.king@stanford.edu	N	05/27/2015	N/A	N/A

Title: Promoting Healthy Lifestyles Using Mobile Phones
Approval Period: 07/02/2015 - 06/30/2016

13. Consent Background

13.1 Waiver of Documentation MILES Phone Screener ENGLISH

Sponsor's Consent Version Number: (if any):

a) Describe the informed consent process. Include the following.

- **i) Who is obtaining consent? (The person obtaining consent must be knowledgeable about the study.)**
- **ii) When and where will consent be obtained?**
- **iii) How much time will be devoted to consent discussion?**
- **iv) Will these periods provide sufficient opportunity for the participant to consider whether or not to participate and sign the written consent?**
- **v) What steps are you taking to minimize the possibility of coercion and undue influence?**
- **vi) If consent relates to children and if you have a reason for only one parent signing, provide that rationale for IRB consideration.**

Most participants for this study will be screened using an on-line eligibility screening survey. In the infrequent event that a participant is unable to complete the n-line screener, trained research staff will screen individuals for eligibility to participate in the study over the telephone. Consent to proceed with the eligibility screening over the phone will be gathered after the participant has been read the telephone screening script which explains the purpose and requirements of the study in great detail. This should provide sufficient opportunity for the participant to decide to participate in the telephone screen. Participants will be informed that all of their responses are voluntary and that they are not required to answer any of the questions. Finally, participants will be informed that there are no consequences if they do not agree to participate in the screen.

b) What is the Procedure to assess understanding of the information contained in the consent? How will the information be provided to participants if they do not understand English or if they have a hearing impairment? See /hrpp/Chapter12.html#ch12_2 HRPP Chapter12.2 for guidance.

Participants will be asked to describe the study purpose and procedures to ensure understanding of the information that was conveyed to them over the telephone screen.

c) What steps are you taking to determine that potential participants are competent to participate in the decision-making process? If your study may enroll adults who are unable to consent, describe (i) how you will assess the capacity to consent, (ii) what provisions will be taken if the participant regains the capacity to consent,(iii) who will be used as a legally authorized representative, and (iv) what provisions will be made for the assent of the participant.

Participants have to verbally display their understanding of the telephone screen and the content of the study to ensure they fully understand what they are being asked to participate in.

Select one of the following regulatory criteria for a waiver of documentation (signature) and provide a protocol-specific justification:

**1)**		**45 CFR 46.117(c)(1). For research that is not subject to FDA regulation, the only record linking the participants and the research would be the consent document,and the principal risk would be potential harm resulting from a breach of confidentiality; each participant will be asked whether he/she wants documentation linking the participant with the research, and the participant's wishes govern.**
**2)**	Y	**45 CFR 46.117(c)(2). For research that is not subject to FDA regulation, presents no more than minimal risk of harm to participants and involves no procedures for which written consent is normally required outside of the research context.**
**2)**		**21 CFR 56.109(c)(1). For research that is subject to FDA regulation, presents no more than minimal risk of harm to participants and involves no procedures for which written consent is normally required outside of the research context.**

Rationale for above selection:

The purpose of the telephone screen is to collect minimal information to determine if the participant

meets minimal eligibility criteria for the study.

13.2 Alteration of Consent **2014-2015 ICF Intervention Trial - English**

Sponsor's Consent Version Number: (if any):

a) Describe the informed consent process. Include the following.

- **i) Who is obtaining consent? (The person obtaining consent must be knowledgeable about the study.)**
- **ii) When and where will consent be obtained?**
- **iii) How much time will be devoted to consent discussion?**
- **iv) Will these periods provide sufficient opportunity for the participant to consider whether or not to participate and sign the written consent?**
- **v) What steps are you taking to minimize the possibility of coercion and undue influence?**
- **vi) If consent relates to children and if you have a reason for only one parent signing, provide that rationale for IRB consideration.**

Prior to agreeing to participate in the study, interested individuals are all oriented to the study in a session that lasts approximately 45 minutes. At the orientation, the study is explained to potential participants in detail and they are given the opportunity to ask questions. If after attending the orientation session, individuals wish to participate then a trained researcher who is knowledgeable about the study goes through the informed consent form with the participant and describes the risks and benefits, the time involvement, emphasizes that there is no payment for participating in this research, informs participants of their rights and points out the contact information provided in the informed consent document. This process will take approximately 15 minutes. The 1 hour provided for the orientation session and the review of the informed consent documentation will provide sufficient opportunity for participants to consider whether or not to participate and will minimize the possibility of coercion and undue influence.

b) What is the Procedure to assess understanding of the information contained in the consent? How will the information be provided to participants if they do not understand English or if they have a hearing impairment? See /hrpp/Chapter12.html#ch12_2 HRPP Chapter12.2 for guidance.

Participants will be asked to repeat back the purpose of the study and the requirements for participation. This method has proven successful in identifying individuals who may not understand English or have a hearing impairment.

c) What steps are you taking to determine that potential participants are competent to participate in the decision-making process? If your study may enroll adults who are unable to consent, describe (i) how you will assess the capacity to consent, (ii) what provisions will be taken if the participant regains the capacity to consent,(iii) who will be used as a legally authorized representative, and (iv) what provisions will be made for the assent of the participant.

Requesting that participants repeat back the purpose of the study and the study procedures requires the level of comprehension and capacity necessary to participate in the decision-making process. Participants unable to consent will not be enrolled.

Address the following four regulatory criteria for an alteration of consent and provide protocol-specific justification for each:

1) Y **The research involves no more than minimal risk to the participants.**

The following intervention trial involves no more than minimal risk to participants. Participants will be prescribed to gradually increase their physical activity levels and decrease their sedentary behavior. The increases in physical activity will be based on Dr. King's group's experience with over 20 years worth of work promoting increased physical activity. For participants in the social application, confederates will be used as part of the experimental manipulation, in part, to ensure appropriately motivational but achievable social norms are established that will inspired increased physical activity but will not be too high and thus have the potential to inspire over-exertion and injury of participants.

2) Y **The waiver or alteration will not adversely affect the rights and welfare of the participants.**

The waiver of alteration will not adversely affect the rights of the participants. The use of confederates in the social app does not have any impact on the rights of the participants. With regard to welfare, the confederates in this study, if anything, will likely improve the welfare of participants as it will allow research personnel to manipulate the social norms and moderate messages being sent back and forth over the message board to ensure appropriate social norms and content.

3) Y **The research could not practically be carried out with out the waiver or alteration.**

Based on previous research, establishing appropriate social norms that are slightly high but achievable is vital to establishing proper motivation for behavior change. Particularly since we are recruiting individuals that are starting at low levels of physical activity, we will not have appropriate role models for physical activity and sedentary behavior in the study for establishing appropriate social norm. Based on this and also the need to deliver an active intervention to the first few participants and to spur appropriate content within the message board, the confederates are required for the study to properly test the impact of a "social" intervention for promoting increased physical activity and decreased sedentary behaviors under optimal conditions.

4) Y **Whenever appropriate, the participants will be provided with additional pertinent information after participation.**

The debriefing will be conducted immediately after the completion of the study and will be provided by research personnel who are well-informed about the research project and the deception. Research personnel will follow the debriefing script and also provide the debriefing statement to all participants in the social application.

13.3 Alteration of Consent 2014-2015 ICF Intervention Trial - Spanish

Sponsor's Consent Version Number: (if any):

a) Describe the informed consent process. Include the following.

i) Who is obtaining consent? (The person obtaining consent must be knowledgeable about the study.)

ii) When and where will consent be obtained?

iii) How much time will be devoted to consent discussion?

iv) Will these periods provide sufficient opportunity for the participant to consider whether or not to participate and sign the written consent?

v) What steps are you taking to minimize the possibility of coercion and undue influence?

vi) If consent relates to children and if you have a reason for only one parent signing, provide that rationale for IRB consideration.

Prior to agreeing to participate in the study, interested individuals are all oriented to the study in a session that lasts approximately 45 minutes. At the orientation, the study is explained to potential participants in detail and they are given the opportunity to ask questions. If after attending the orientation session, individuals wish to participate then a trained researcher who is knowledgeable about the study goes through the informed consent form with the participant and describes the risks and benefits, the time involvement, emphasizes that there is no payment for participating in this research, informs participants of their rights and points out the contact information provided in the informed consent document. This process will take approximately 15 minutes. The 1 hour provided for the orientation session and the review of the informed consent documentation will provide sufficient opportunity for participants to consider whether or not to participate and will minimize the possibility of coercion and undue influence.

b) What is the Procedure to assess understanding of the information contained in the consent? How will the information be provided to participants if they do not understand English or if they have a hearing impairment? See /hrpp/Chapter12.html#ch12_2 HRPP Chapter12.2 for guidance.

Participants will be asked to repeat back the purpose of the study and the requirements for participation. This method has proven successful in identifying individuals who may not understand English or have a hearing impairment.

c) What steps are you taking to determine that potential participants are competent to participate in the decision-making process? If your study may enroll adults who are unable to consent, describe (i) how

you will assess the capacity to consent, (ii) what provisions will be taken if the participant regains the capacity to consent,(iii) who will be used as a legally authorized representative, and (iv) what provisions will be made for the assent of the participant.

Requesting that participants repeat back the purpose of the study and the study procedures requires the level of comprehension and capacity necessary to participate in the decision-making process. Participants unable to consent will not be enrolled.

Address the following four regulatory criteria for an alteration of consent and provide protocol-specific justification for each:

1) Y **The research involves no more than minimal risk to the participants.**

The following intervention trial involves no more than minimal risk to participants. Participants will be prescribed to gradually increase their physical activity levels and decrease their sedentary behavior. The increases in physical activity will be based on Dr. King's group's experience with over 20 years worth of work promoting increased physical activity. For participants in the social application, confederates will be used as part of the experimental manipulation, in part, to ensure appropriately motivational but achievable social norms are established that will inspired increased physical activity but will not be too high and thus have the potential to inspire over-exertion and injury of participants.

2) Y **The waiver or alteration will not adversely affect the rights and welfare of the participants.**

The waiver of alteration will not adversely affect the rights of the participants. The use of confederates in the social app does not have any impact on the rights of the participants. With regard to welfare, the confederates in this study, if anything, will likely improve the welfare of participants as it will allow research personnel to manipulate the social norms and moderate messages being sent back and forth over the message board to ensure appropriate social norms and content.

3) Y **The research could not practically be carried out with out the waiver or alteration.**

Based on previous research, establishing appropriate social norms that are slightly high but achievable is vital to establishing proper motivation for behavior change. Particularly since we are recruiting individuals that are starting at low levels of physical activity, we will not have appropriate role models for physical activity and sedentary behavior in the study for establishing appropriate social norm. Based on this and also the need to deliver an active intervention to the first few participants and to spur appropriate content within the message board, the confederates are required for the study to properly test the impact of a "social" intervention for promoting increased physical activity and decreased sedentary behaviors under optimal conditions.

4) Y **Whenever appropriate, the participants will be provided with additional pertinent information after participation.**

The debriefing will be conducted immediately after the completion of the study and will be provided by research personnel who are well-informed about the research project and the deception. Research personnel will follow the debriefing script and also provide the debriefing statement to all participants in the social application.

13.4 Consent — 2014-2015 Consent for Intervention - English

Sponsor's Consent Version Number: (if any):

a) **Describe the informed consent process. Include the following.**

- **i) Who is obtaining consent? (The person obtaining consent must be knowledgeable about the study.)**
- **ii) When and where will consent be obtained?**
- **iii) How much time will be devoted to consent discussion?**
- **iv) Will these periods provide sufficient opportunity for the participant to consider whether or not to participate and sign the written consent?**
- **v) What steps are you taking to minimize the possibility of coercion and undue influence?**

Title: Promoting Healthy Lifestyles Using Mobile Phones
Approval Period: 07/02/2015 - 06/30/2016

vi) If consent relates to children and if you have a reason for only one parent signing, provide that rationale for IRB consideration.

Consent will be obtained by a member of the research team knowledgeable about the study and with prior consenting experience. The consent process will include a discussion of the study procedures and requirements for participation as well as a review of participant rights and resources for consent. These issues will be discussed specifically to highlight the factors relevant to their choice to participate to minimize coercion.

b) What is the Procedure to assess understanding of the information contained in the consent? How will the information be provided to participants if they do not understand English or if they have a hearing impairment? See /hrpp/Chapter12.html#ch12_2 HRPP Chapter12.2 for guidance.

Participants will be asked to repeat back the purpose of the study and the requirements for participation. A research assistant who is fluent in Spanish will be available to answer any questions participants may have.

c) What steps are you taking to determine that potential participants are competent to participate in the decision-making process? If your study may enroll adults who are unable to consent, describe (i) how you will assess the capacity to consent, (ii) what provisions will be taken if the participant regains the capacity to consent,(iii) who will be used as a legally authorized representative, and (iv) what provisions will be made for the assent of the participant.

Consent will be obtained by a member of the research team knowledgeable about te study and with prior consenting experience. Participants will be asked to repeat back the purpose of the study and the requirements for participation. A research assistant who is fluent in Spanish will be available to answer any questions participants may have.

13.5 Consent — 2014-2015 Consent for Intervention - Spanish

Sponsor's Consent Version Number: (if any):

a) Describe the informed consent process. Include the following.

i) Who is obtaining consent? (The person obtaining consent must be knowledgeable about the study.)

ii) When and where will consent be obtained?

iii) How much time will be devoted to consent discussion?

iv) Will these periods provide sufficient opportunity for the participant to consider whether or not to participate and sign the written consent?

v) What steps are you taking to minimize the possibility of coercion and undue influence?

vi) If consent relates to children and if you have a reason for only one parent signing, provide that rationale for IRB consideration.

Consent will be obtained by a member of the research team knowledgeable about the study and with prior consenting experience. The consent process will include a discussion of the study procedures and requirements for participation as well as a review of participant rights and resources for consent. These issues will be discussed specifically to highlight the factors relevant to their choice to participate to minimize coercion.

b) What is the Procedure to assess understanding of the information contained in the consent? How will the information be provided to participants if they do not understand English or if they have a hearing impairment? See /hrpp/Chapter12.html#ch12_2 HRPP Chapter12.2 for guidance.

Participants will be asked to repeat back the purpose of the study and the requirements for participation. A research assistant who is fluent in Spanish will be available to answer any questions participants may have.

c) What steps are you taking to determine that potential participants are competent to participate in the decision-making process? If your study may enroll adults who are unable to consent, describe (i) how you will assess the capacity to consent, (ii) what provisions will be taken if the participant regains the capacity to consent,(iii) who will be used as a legally authorized representative, and (iv) what provisions will be made for the assent of the participant.

Consent will be obtained by a member of the research team knowledgeable about the study and with prior consenting experience. Participants will be asked to repeat back the purpose of the study and the requirements for participation. A research assistant who is fluent in Spanish will be available to answer any questions participants may have.

Title: Promoting Healthy Lifestyles Using Mobile Phones
Approval Period: 07/02/2015 - 06/30/2016

13.6 Waiver of Documentation — Eligibility screener Spanish 2013

Sponsor's Consent Version Number: (if any):

a) Describe the informed consent process. Include the following.

- **i) Who is obtaining consent? (The person obtaining consent must be knowledgeable about the study.)**
- **ii) When and where will consent be obtained?**
- **iii) How much time will be devoted to consent discussion?**
- **iv) Will these periods provide sufficient opportunity for the participant to consider whether or not to participate and sign the written consent?**
- **v) What steps are you taking to minimize the possibility of coercion and undue influence?**
- **vi) If consent relates to children and if you have a reason for only one parent signing, provide that rationale for IRB consideration.**

Potential participants will be screened to see if they are eligible to participate in the study by research staff who are knowledgeable about the study and who have been trained in the protection of human subjects in research. The screening process will be done either over the telephone or in person at the recruitment site at a time convenient to both the potential participants and the researchers. If at the recruitment site, the eligibility screener will be done in a private place. Enough time will be provided to explain the purpose of the screener and the study protocol and answer any questions potential participants have. This should take approximately 30 minutes. Eligible participants will be more thoroughly oriented to the study on a separate occasion and only after the study has been fully described and the eligible participants have had an opportunity to ask questions will written informed consent be gathered. Participants will be informed that all of their responses are voluntary and that they are not required to answer any of the questions. Participants will be informed that there are no consequences if they do not agree to participate in the eligibility screener.

b) What is the Procedure to assess understanding of the information contained in the consent? How will the information be provided to participants if they do not understand English or if they have a hearing impairment? See /hrpp/Chapter12.html#ch12_2 HRPP Chapter12.2 for guidance.

Participants will verbally provide their understanding of the process and purpose of the eligibility screening. A bilingual English/Spanish researcher will administer the eligibility screener. The eligibility screener will also be available in Spanish for potential participants to read if necessary.

c) What steps are you taking to determine that potential participants are competent to participate in the decision-making process? If your study may enroll adults who are unable to consent, describe (i) how you will assess the capacity to consent, (ii) what provisions will be taken if the participant regains the capacity to consent,(iii) who will be used as a legally authorized representative, and (iv) what provisions will be made for the assent of the participant.

Participants will verbally provide their understanding of the process and purpose of the eligibility screening and the study protocol. Only participants who exhibit understanding of the eligibility screener and the study protocol and are able to consent will complete the eligibility screener. A bilingual English/Spanish researcher will administer the eligibility screener.

Select one of the following regulatory criteria for a waiver of documentation (signature) and provide a protocol-specific justification:

1)		**45 CFR 46.117(c)(1). For research that is not subject to FDA regulation, the only record linking the participants and the research would be the consent document,and the principal risk would be potential harm resulting from a breach of confidentiality; each participant will be asked whether he/she wants documentation linking the participant with the research, and the participant's wishes govern.**
2)		**45 CFR 46.117(c)(2). For research that is not subject to FDA regulation, presents no more than minimal risk of harm to participants and involves no procedures for which written consent is normally required outside of the research context.**
2)	Y	**21 CFR 56.109(c)(1). For research that is subject to FDA regulation, presents no more than minimal risk of harm to participants and involves no procedures for which written consent is normally required outside of the research context.**

Rationale for above selection:

The purpose of the eligibility screener is to collect minima information to determine if the participant meets eligibility criteria for inclusion in the study.

14. Assent Background (less than 18 years of age)

15. HIPAA Background

15.1 Authorization — **hippa authorization form**

15.2 Waiver of Authorization for Recruitment — **promoting healthy lifestyles using mobile phones**

a) **Describe the protected health information (PHI) needed to conduct screening or recruitment. PHI is health information linked to HIPAA identifiers (see section 11). List BOTH health information AND HIPAA identifiers. If you are using STRIDE, use the Clinical Data Work Sheet to ensure that your request will match your IRB-approved protocol. Be consistent with information entered in section 11b.**

Name, telephone number and mailing address will be collected during the telephone screen for future contact with potential participants. The telephone screen will also collect and record birth date and limited health information to determine age and medical eligibility (i.e., ability to initiate a program of physical activity) for study participation (Please see the "telephone screen form" attached).

b) Please Answer:

Y **Do you certify that the use or disclosure of protected health information involves no more than a minimal risk to the privacy of individuals?**

Y **Do you certify that the research could not practically be conducted with out the waiver?**

Y **Do you certify that you have adequate written assurances that the protected health information will not be reused or disclosed to any other person or entity, except as required by law, for authorized oversight of the research project, or for other research for which the use or disclosure of protected health information would be permitted?**

Y **Do you certify that the research could not practically be conducted with out access to and use of the protected health information?**

c) **Please describe an adequate plan to protect any identifiers from improper use and disclosure.**

No personally identifiable information is expected to be shared. The identity of participants will not be disclosed in any reports or publications. All data will be free of any identifying information and will be coded by ID number. A master list of ID numbers and identifying information will be kept separate from the data in both physical location (locked cabinet) and in electronic datasets (password protected computer). Only research staff that has a study related need for identifying information will be allowed access.

d) **Please describe an adequate plan to destroy the identifiers at the earliest opportunity consistent with conduct of the research, unless there is a health or research justification for retaining the identifiers or such retention is otherwise required by law.**

Title: Promoting Healthy Lifestyles Using Mobile Phones
Approval Period: 07/02/2015 - 06/30/2016

PHI recorded information will be destroyed upon completion of the study (once manuscripts have been published).

16. Attachments

Attachment Name	Attached Date	Attached By	Submitted Date
Baseline and Follow up questionnaires	08/19/2009	ehekler	
Baseline and Follow up Interview Scripts	08/19/2009	ehekler	
Daily Questionnaire Packet	08/19/2009	ehekler	
Physical Activity Interview-Form	08/19/2009	ehekler	
SPO 45762 - NIH	08/20/2009	jlarsen	
Focus Group Telephone Screen Form	09/14/2009	ehekler	
Focus Group Protocol/Questions	09/14/2009	ehekler	
Focus Group Poster	09/14/2009	ehekler	
Focus Group Newspaper Advertisement	09/14/2009	ehekler	
User Testing Questionnares and Description of Apps	05/28/2010	ehekler	
User Testing Description for the Andriod Market	05/28/2010	ehekler	
Affect app "story board" (i.e.,intervention desc.)	10/23/2010	ehekler	
Social App "storyboard" (i.e., intervention desc.)	10/23/2010	ehekler	
intervention trial newspaper ad	10/25/2010	ehekler	
Intervention Trial Poster	10/25/2010	ehekler	
Intervention trial: Screen Form	10/25/2010	ehekler	
Debriefing Script	10/25/2010	ehekler	
Debriefing Statement-1	11/16/2010	aldeguer	

Recruitment flyer June 2012	06/21/2012	sjwinter	
MILES baseline survey 2012	06/21/2012	sjwinter	
MILES follow up survey 2012	06/21/2012	sjwinter	
MILES eligibility telephone screener 2012	06/21/2012	sjwinter	
MILES eligibility screener response form	06/21/2012	sjwinter	
Focus Group Telephone Screen-1	07/31/2012	jmtanaka	
Online Screening Tool-1	07/31/2012	jmtanaka	
Consent form for Focus Group	07/31/2012	jmtanaka	
User Testing Waiver of Documentation	07/31/2012	jmtanaka	
M.I.Salud Baseline Assessment Espanol_JR 2-25 (1)	02/26/2013	sjwinter	
Eligibility screener - Spanish	02/26/2013	sjwinter	
Orientation powerpoint Spanish	02/26/2013	sjwinter	
Spanish Debriefing Statement	03/13/2013	akfields	
Spanish Debriefing Script	03/13/2013	akfields	
Mi Salud Flyer 4 apps	03/13/2013	akfields	

Obligations

The Protocol Director agrees to:

- Adhere to principles of http://humansubjects.stanford.edu/research/documents/eval_study_designGUI03017.pdf sound scientific research designed to yield valid results
- Conduct the study according to the protocol approved by the IRB
- Be appropriately qualified to conduct the research and be trained in Human Research protection, ethical principles, regulations, policies and procedures
- Ensure all research personnel are adequately trained and supervised

- Ensure that the rights and welfare of participants are protected including privacy and confidentiality of data
- Ensure that, when de-identified materials are obtained for research purposes, no attempt will be made to re-identify them.
- Disclose to the appropriate entities any potential conflict of interest
- Report promptly any new information, modification, or http://humansubjects.stanford.edu/research/documents/Events-Info-Report-to-IRB_GUI03P13.pdf unanticipated problems that raise risks to participants or others
- Apply relevant professional standards.

VA Protocol Directors also certify that:

- All unanticipated internal or local SAEs, whether related or unrelated to the research, will be/have been reported to the IRB
- All subjects entered onto the master list of subjects for the study will sign/have signed an informed consent form prior to undergoing any study interactions or interventions, unless granted a waiver by the IRB.

Any change in the research protocol must be submitted to the IRB for review prior to the implementation of such change. Any complications in participants or evidence of increase in the original estimate of risk should be reported at once to the IRB before continuing with the project. Inasmuch as the Institutional Review Board (IRB) includes faculty, staff, legal counsel, public members, and students, protocols should be written in language that can be understood by all Panel members. The investigators must inform the participants of any significant new knowledge obtained during the course of the research.

IRB approval of any project is for a maximum period of one year. For continuing projects and activities, it is the responsibility of the investigator(s) to resubmit the project to the IRB for review and re-approval prior to the end of the approval period. A Notice to Renew Protocol is sent to the Protocol Director 7 weeks prior to the expiration date of the protocol.

Department Chair must approve faculty and staff research that is not part of a sponsored project. VA applicants must have Division Chief or Ward Supervisor approval. E-mail the Department Chair approval to IRBCoordinator@lists.stanford.edu.

All data including signed consent form documents must be retained for a minimum of three years past the completion of the research. Additional requirements may be imposed by your funding agency, your department, or other entities. (Policy on Retention of and Access to Research Data, Research Policy Handbook, http://doresearch.stanford.edu/policies/research-policy-handbook/conduct-research/retention-and-access-research-data)

PLEASE NOTE: List all items (verbatim) that you want to be reflected in your approval letter (e.g., Amendment, Investigator's Brochure, consent form(s), advertisement, etc.) in the box below. Include number and date when appropriate.

Y By checking this box, I verify that I, as the Protocol Director (PD) responsible for this research protocol, have read and agree to abide by the above obligations, or that I have been delegated authority by the PD to certify that the PD has read and agrees to abide by the above obligations.
